# QTL mapping of flowering time and biomass yield in tetraploid alfalfa (*Medicago sativa* L.)

**DOI:** 10.1186/s12870-019-1946-0

**Published:** 2019-08-16

**Authors:** Laxman Adhikari, Shiva Om Makaju, Ali M. Missaoui

**Affiliations:** Institute of Plant Breeding, Genetics and Genomics and Department of Crop and Soil Sciences, The University of Georgia, Athens, GA USA

**Keywords:** Alfalfa, *Medicago truncatula*, GBS, Single dose allele, Flowering QTL, Biomass

## Abstract

**Background:**

The genetic and genomic basis of flowering time and biomass yield in alfalfa (*Medicago sativa* L.) remains poorly understood mainly due to the autopolyploid nature of the species and the lack of adequate genomic resources. We constructed linkage maps using genotyping-by-sequencing (GBS) based single dose allele (SDA) SNP and mapped alfalfa timing of flowering (TOF), spring yield (SY), and cumulative summer biomass (CSB) in a pseudo-testcross F1 population derived from a fall dormant (3010) and a non-dormant (CW 1010) cultivars. We analyzed the quantitative trait loci (QTL) to identify conserved genomic regions and detected molecular markers and potential candidate genes associated with the traits to improve alfalfa and provide genomic resources for the future studies.

**Results:**

This study showed that both fall dormant and non-dormant alfalfa cultivars harbored QTL for early and late flowering, suggesting that flowering time in alfalfa is not an indicator of its fall dormancy (FD) levels. A weak phenotypic correlation between the flowering time and fall dormancy (FD) in F1 and checks also corroborated that alfalfa FD and TOF are not the predictors of one another. The relationship between flowering time and alfalfa biomass yield was not strong, but the non-dormant had relatively more SY than dormant. Therefore, selecting superior alfalfa cultivars that are non-dormant, winter-hardy, and early flowering would allow for an early spring harvest with enhanced biomass. In this study, we found 25 QTL for TOF, 17 for SY and six QTL for CSB. Three TOF related QTL were stable and four TOF QTL were detected in the corresponding genomic locations of the flowering QTL of *M. truncatula*, an indication of possible evolutionarily conserved regions. The potential candidate genes for the SNP sequences of QTL regions were identified for all three traits and these genes would be potential targets for further molecular studies.

**Conclusions:**

This research showed that variation in alfalfa flowering time after spring green up has no association with dormancy levels. Here we reported QTL, markers, and potential candidate genes associated with spring flowering time and biomass yield of alfalfa, which constitute valuable genomic resources for improving these traits via marker-assisted selection (MAS).

**Electronic supplementary material:**

The online version of this article (10.1186/s12870-019-1946-0) contains supplementary material, which is available to authorized users.

## Background

In alfalfa (*Medicago sativa* L.), timing of flowering (TOF) is important for the completion of reproduction and adaptation to the environment [1]. Alfalfa TOF also serves as a guideline for harvesting time as farmers often cut alfalfa at the early bloom stage (http://extension.uga.edu/publications/detail.html?number=B1350&title=Alfalfa%20Management%20in%20Georgia.). Harvesting at the proper stage helps to balance forage quality, yield, and maintaining healthy stubbles for the future stand (https://crops.extension.iastate.edu/cropnews/2010/05/when-make-first-spring-cut-alfalfa-and-mixed-alfalfagrass.). After the first clipping, the following blooming stage comes in about 28–35 days (http://extension.uga.edu/publications/detail.html?number=B1350&title=Alfalfa%20Management%20in%20Georgia.). Unlike some other forages, harvesting before reaching the full seed stage is common in alfalfa for high total digestible nutrients (TDN) which decreases with maturity stage. Early vs. late flowering are two important considerations for breeders while working on flowering date. In unfavorable climates where drought or heat stresses are major concerns for crop production, early flowering could be a desirable trait because of the short growing season [2]. Genotypes that flower early in the spring could be valuable for filling the seasonal forage gap that exists due to winter dormancy. On the other hand, delayed flowering can also be a desirable trait to minimize damage from abiotic stresses as well as to enhance biomass yield via longer vegetative growth [3]. For instance, in bioenergy crops, such as switchgrass and elephant grass, late flowering increases biomass accumulation. Similarly, delayed flowering in alfalfa could be desirable for protecting the plants from late winter and early spring frost in addition to enhanced biomass yield. However, delayed flowering may be associated with lower forage quality. The flower bud initiation to seed pod formation stages in alfalfa are largely dependent on the environment. Photoperiod and temperature have great impacts on alfalfa flowering time and the underlying genetic factors are important to manipulate this trait (https://www.alfalfa.org/pdf/HowAnAlfalfaPlantDevelops.pdf.).

The genetic and genomic basis of flowering time has been investigated extensively in cereals and row crops, whereas such information is scarce in herbaceous perennials. QTL and candidate genes associated with TOF were reported in several plant species, such as Arabidopsis [4], wheat [5], rice [6], and maize [7]. In Arabidopsis, three distinct genetic pathways; long-day, autonomous, and gibberellins were reported for flowering time control [8]. Vernalization was also reported as a mechanism associated with flowering time in Arabidopsis. Legumes such as pea (*Pisum sativum*), soybean (*Glycine max* L.) and *M. truncatula* have been investigated for the genetic basis of variation in TOF. Pierre et al. (2008) found QTL for flowering date in three mapping populations of *M. truncatula* on chromosome seven [1]. The FT family florigen (MtFTa1, MtFTb1 and MtFTc) detected for *M. truncatula* flowering trait successfully complemented the late-flowering Arabidopsis *ft-1* mutant plants and induced early flowering [9]. Similarly, involvement of some genes such as MsLFY [10], CONSTANS-LIKE [11], SPL13 [12], MsZFN [13], MsFRI-L [14], and *MsFRI-L* in alfalfa flowering time variation have been described using the reverse genetics approaches like molecular cloning and gene expression. However, single gene expression analysis with knockouts or transgenics seems insufficient to account for the extensive quantitative variation in the population [15], which can be explained using QTL mapping with a high-resolution genetic map.

The high biomass yield is one of the key considerations for market acceptance of a newly developed alfalfa cultivar. For a cool season crop like alfalfa, biomass yields after spring regrowth and in subsequent harvests account for a remarkable portion of the year-round production. Spring is one of the most favorable season for alfalfa growth as the optimum temperature for its growth is 15–25 °C which is a characteristic to most temperate regions [16]. Alfalfa spring yield (SY) also correlates with other important characteristics such as FD [17]. Non-dormant genotypes often start regrowth early in the spring, flower early, generate high biomass, and start quick shoot regrowth after harvesting even in subsequent summer months compared to dormant genotypes [17]. Faster spring growth is also a positive indicator of higher summer growth [18]. The identification of genomic regions controlling spring biomass yield enables the understanding of the genetic basis of the trait and the utilization of the associated markers in molecular breeding to enhance biomass yield in alfalfa.

QTL mapping in alfalfa using traditional markers and phenotypic data based on plant vigor, height, canopy-width and canopy-density enabled the detection of some forage biomass related QTL [19]. Alfalfa biomass associated marker loci were previously reported using RFLP and simple sequence repeat (SSR) markers [20]. Li et al. (2011) identified 15 SSR markers strongly associated (*P* < 0.005) with yield in an alfalfa breeding population [21]. Some QTL having phenotypic effects up to 6% and associated with alfalfa biomass in drought stress conditions were reported [22]. A GWAS study reported SNPs associated with biomass yield of a diploid alfalfa (*M. falcata*) population corresponding to the genomic regions of *M. truncatula* genes for early growth, meristem development, and cell growth/division [23]. Biomass related consensus genomic regions were identified in multiple *M. truncatula* populations [24]. Li et al. (2015) conducted genomic selection (GS) in alfalfa using GBS markers and phenotypic selection for 2 years illustrating the potential of the GS method in enhancing genetic gain for yield in alfalfa [25]. The GS for alfalfa yield conducted using different reference populations exhibited moderate prediction accuracy and the method was efficient [26].

Tetraploid alfalfa has tetrasomic inheritance and the loci segregate with complex patterns in subsequent generations. Therefore, only certain biallelic markers segregating in specific patterns are usable for constructing genetic linkage maps of tetraploid alfalfa using software designed for diploid species [27]. Linkage mapping in autopolyploids is routinely performed with F1 populations derived from two heterozygous parents using single dose allele (SDA) markers unique to each parent and segregating in 1:1 (Aaaa x aaaa) [27]. This segregation ratio is similar to a testcross (1:1), thus the name pseudo-testcross is used for this linkage mapping strategy [28]. Using this strategy, linkage maps can be constructed by the software designed for diploids such as JoinMap (https://www.kyazma.nl/docs/JM5Manual.pdf). Although the pseudo-testcross strategy uses only a portion of markers of the genome, it is still useful for polyploid species with complex genomes and marker segregation ratios, and the method has been successfully used in various species [28, 29].

Identifying significant marker(s) and QTL of target trait allows the introgression of desired alleles into elite germplasm for increasing the number of favorable alleles [24]. To our knowledge, the alfalfa adaptations and agronomic traits we mapped in this study have not been well studied or mapped on low saturation genetic linkage maps. Mapping these traits on a higher resolution genetic map will result in the accurate detection of genomic positions of associated loci and open the door for functional analyses of these genomic regions. Furthermore, the knowledge of correlations among various alfalfa traits and their genetic basis may allow simultaneous improvement of traits to increase biomass and forage quality. Therefore, the objectives of this study were: (i) to identify QTL controlling alfalfa timing of flowering (TOF), spring yield (SY), and cumulative summer biomass (CSB), (ii) to evaluate the phenotypic relationship between various alfalfa adaptation and agronomic traits, and (iii) to search potential candidate genes related to these traits.

## Results

### Phenotypic assessment, G x E and heritability

**Fig. 1:**
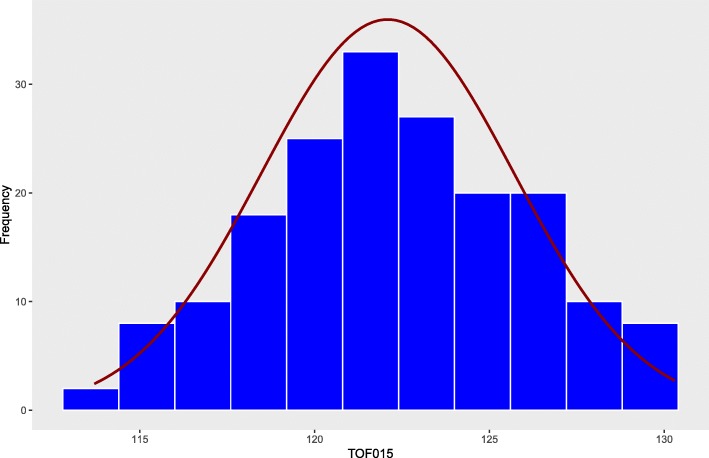
Distribution of timing of flowering (TOF) among alfalfa bi-parental (3010 x CW 1010) hybrid population in JPC environment in spring 2015 calculated as LS means of days to the flowering. There were 181 F1 genotypes which showed near to the normal distribution for the TOF. X-axis represents the LS means of TOF and Y-axis represents the genotype frequency for the corresponding TOF

There were significant differences (*P* < 0.05) in TOF among F1 individuals in all environments and years (Table 2). The F1 population exhibited near normal distribution for each dataset (Fig. 1). The least squares mean (LS mean) estimated for TOF at the J. Phil Campbell Sr. Research and Education Center (JPC) in Watkinsville ranged from 112 to 130 Julian calendar days and from 85 to 110 days for the years 2015 and 2017, respectively (Table 2). In the Georgia Mountain Research and Education Center in Blairsville (BVL), the LS means for TOF varied from 146 to 163 days and 118 to 136 days for the years 2015 and 2017, respectively (Table 2). The parent CW 1010 and 3010 did not show significant differences (*P* ≥ 0.05) in their TOF means in two-sample t-test (SAS 9.4, SAS Institute, Cary, NC, USA). The dormant parent 3010 had slightly shorter average flowering days than the non-dormant parent CW 1010 (Table 2). The non-dormant parent CW 1010 had a substantial amount of regrowth in winter and sustained winter injury compared to the dormant 3010, which possibly influenced the flowering time. Abundant transgressive segregants were present on both early and late flowering sides of the distribution.

Variation among F1 individuals was observed for the spring yield (SY) and for the three subsequent summer cuts (Table 2). The F1 progenies were significantly different (*P* < 0.05) for both SY and summer cuts as revealed by the analysis of variance (ANOVA). At the JPC, the F1 LS means estimated for SY in 2017 ranged from 0.33 to 1.9 kg/plant (Table 2). Similarly, in BVL, the LS means for F1 individuals ranged from 0.25 to 2.05 kg/plant for SY in 2017. The LS means for SY for F1 exhibited near normal distribution for the datasets recorded in both environments. The dry matter percentages were about 30% in the spring biomass harvest at both environments and about 25% in the summer biomass.

The effects of genotype (G), environment (E) and their interaction (G x E) were significant (*P* < 0.05) for each of the traits analyzed (Table 1). Variance components and heritability of each alfalfa trait investigated here were calculated based on datasets, for each year and each environment. The broad-sense heritability (H^2^) of TOF ranged from 0.38–0.75 whilst the H^2^ of spring biomass yield varied from 0.18 to 0.75 (Table 1). Mean squares of genotypes and G x E obtained from the variance analysis were also reported (Table 1). Since we could not collect spring yield in BVL in 2018, the mean squares for SY018 dataset is not available.

**Table 1:**
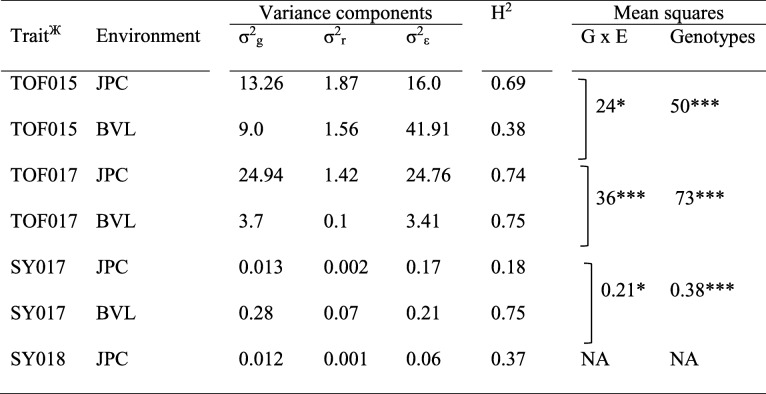
Variance components and broad-sense heritability (H^2^) of the timing of flowering (TOF) and the spring yield (SY) for two locations

The two locations included the J. Phil Campbell Sr. Research and Education Center (JPC) in Watkinsville and the Georgia Mountain Research and Education Center (BVL) in Blairsville

NA = data not available or available for only one environment

H^2^ = Broad-sense heritability, σ^2^_g_ = Genotypic variance, σ^2^_r_ = Between block variance, σ^2^_ε_ = Variance component of residual error, *TOF* Timing of flowering, *SY* Spring yield

*P < 0.05, ** *P* < 0.001

^Ж^Suffix on traits: 015, 017 and 018 indicate the year of data collection

### Correlations between TOF and other traits

A weak negative phenotypic correlation was observed between TOF and FD in the alfalfa F1 population, indicating that the genotypes with shorter days to flowering have a higher dormancy rating or are non-dormant (Tables 3 and 4). The correlation coefficient (*r*) values calculated for selected phenotypic traits are given in Tables 3 and 4. In the JPC environment, the highest correlation coefficient (*r* = − 0.44, *P* < 0.01) between TOF and dormancy was observed for datasets TOF017 and WD017. However, FD recorded in the fall (FD015 and FD016) showed a weaker relationship with TOF. Also, in BVL, the *r* values calculated for TOF and FD exhibited a weak negative to non-significant (*P* ≥ 0.05) correlations (Table 4). The highest correlation between FD and TOF for this environment was (*r* = − 0.13, *P* < 0.05) (Table 4). We also analyzed the FD check cultivars for the TOF and FD relationship (data not given), but we could not observe any strong correlations between these traits. Further, no significant difference (*P* ≥ 0.05) was observed in TOF LS means estimated for dormant and non-dormant parents (Table 2). This suggests that the spring TOF is not a good predictor of fall dormancy in alfalfa. However, the relationship may be specific to this mapping population, which was derived from adapted dormant and non-dormant cultivars.

**Table 2:** Least square (LS) means for F1 and the parents for the timing of flowering (TOF), the spring yield (SY) and the cumulative summer biomass (CSB)

Trait	Environment	F1^a^	3010^b^	CW 1010^b^
TOF015	JPC	112–130	117	119
TOF017	JPC	85–110	101	102
TOF015	BVL	146–163	149	151
TOF017	BVL	118–136	118	121
SY017	JPC	0.33–1.9	0.64	1.05
CSB	JPC	0.11–1.48	0.347	0.34
SY018	JPC	0.10–1.35	0.83	0.86
SY017	BVL	0.25–2.05	1.08	0.7
CSB	BVL	0.05–0.75	0.37	0.25

The units for TOF, SY, and CSB are in Julian days, Kg plant^−1^, and Kg plant^−1^, respectively

The LS means are presented as the range for F1 and as absolute means for the parents (3010 and CW 1010). The CSB is the cumulative summer biomass yield harvested in 3 subsequent cuts

^a^Range of LS means of trait in F1 progeny; ^b^Parental mean for traits under given environment and year. The two locations included the J. Phil Campbell Sr. Research and Education Center (JPC) in Watkinsville and the Georgia Mountain Research and Education Center (BVL) in Blairsville

TOF in the alfalfa F1 population at JPC exhibited a weak negative correlation with the WH scores (Table 3). However, the correlation values are mostly non-significant in BVL for TOF and WH except a significant weak negative correlation between TOF015 and WH016. The negative correlation between TOF and WH at JPC suggests that the winter-hardy plants (lower WH score) reached maximum flowering later in the spring. The highest correlation value (*r* = − 0.43, *P* < 0.01) was observed between TOF017 and WH017 (Table 3) at the JPC location. However, in the BVL environment the correlation between TOF and WH was non-significant (*P* ≥ 0.05). Although the correlation observed between TOF and WH was weak, it seemed that the winter-hardy plants may need longer time to reach the flowering stage. Weak negative (*r* = − 0.24, *P* < 0.05) to non-significant (*P* ≥ 0.05) correlations were observed between TOF and spring yield (SY) in both environments (Tables 3 and 4). Similar correlations were observed between TOF and CSB.

**Table 3:** Correlations between different phenotypic traits evaluated in an F1 pseudo-testcross mapping population at the JPC

	TOF015	TOF017	SY017	CSB	SY018	FD015	FD016	WD017	WH016	WH017
TOF015		0.33**	− 0.04^NS^	− 0.06^NS^	− 0.04^NS^	− 0.19**	− 0.11*	− 0.28**	− 0.22**	− 0.21**
TOF017			− 0.17**	− 0.12**	− 0.02^NS^	− 0.16**	− 0.28**	− 0.44**	− 0.33**	− 0.43**
SY017				0.35**	0.36**	0.18**	0.18**	0.29**	0.13**	0.20**
CSB					0.18**	0.05^NS^	0.01^NS^	0.07^NS^	0.06^NS^	0.04^NS^
SY018						0.24**	0.17**	0.26**	0.15*	0.14**

JPC refers to the J. Phil Campbell Sr. Research and Education Center (JPC) in Watkinsville, GA

*P < 0.05, **P < 0.01, ^NS^ non-signifiant

**Table 4:** Correlations between different phenotypic traits evaluated in an F1 pseudo-testcross mapping population at the BVL

	TOF015	TOF017	SY017	CSB	FD015	FD016	WD017	WH016	WH017
TOF015		− 0.05^NS^	− 0.03^NS^	− 0.03^NS^	− 0.10*	− 0.11*	− 0.05^NS^	− 0.11*	− 0.02^NS^
TOF017			− 0.24**	− 0.26**	− 0.13*	− 0.06^NS^	− 0.07^NS^	− 0.06^NS^	− 0.06^NS^
SY017				0.37**	0.30**	0.15*	0.12*	− 0.13*	− 0.06^NS^
CSB					0.11*	0.01^NS^	− 0.01^NS^	− 0.01^NS^	− 0.16**

BVL refers to the Georgia Mountain Research and Education Center (BVL) in Blairsville

*TOF* Timing of flowering, *SY* Spring yield, *FD* Fall dormancy, *WD* Winter dormancy, *WH* Winter hardiness and, *CSB* Cumulative summer biomass

*P < 0.05, **P < 0.01, ^NS^non-signifiant

### Correlation between FD and SY

Dormancy assessed in the fall (FD015, FD016) and winter (WD017) displayed significant (*P* < 0.05) positive correlations with SY (SY017 and SY018), suggesting that a non-dormant alfalfa genotype has higher yield even in the spring (Tables 3 and 4). In the JPC environment, the significant correlation (*r* = 0.29, *P* < 0.01) was observed between WD017 and SY017, while in BVL, the correlation coefficient (r) up to 0.30 (*P* < 0.01) was obtained between FD015 and SY017. The CSB over three summer cuts did not display significant correlations with FD (Tables 3 and 4). However, CSB and SY showed a weak positive correlation in both environments and years.

### Relationship between WH and SY

The direction of the phenotypic relationship between WH and SY changed with the growing environment. In JPC, a weak positive correlation was observed between WH and SY, indicating that the winter-hardy plants (lower WH score) had a relatively low SY (Table 3). The correlation between WH and SY for JPC population ranged from 0.13 to 0.20 (Table 3). However, in BVL, WH and SY exhibited a weak negative (*r* = − 0.13, *P* < 0.05) to non-significant (*P* ≥ 0.05) correlations. This suggests that the impact of winter damage in BVL environment is higher than in JPC, and the winter-hardy plants had relatively higher spring biomass in that environment.

### TOF and GDD

The plants in BVL flowered approximately 1 month later than those in the JPC. The delayed flowering in BVL is likely due to the lower winter temperatures. In 2015, the average minimum temperature in BVL was − 12.6 °C compared to − 7 °C at JPC (Table 5). In 59 days of two winter months (Jan and Feb) in 2015, the plants in BVL faced < 5 °C (Tb) for 45 days. The winter of 2017 was mild with better winter temperature, which led the plants to flower about 4 weeks earlier than 2015 at both sites (Table 5). Nevertheless, the period of the flowering time difference (about a month) between BVL and JPC did not change that much between the years 2015 and 2017. Therefore, if winter is severe, the first spring harvest of alfalfa will be delayed. The alfalfa population in BVL required higher AGDD than the population in JPC to reach flowering stage (Table 5) regardless of the severity of winter (Table 5). The higher AGDD requirement for BVL plants was most likely due to the longer chilling period in winters with several days having below to near base temperature (Tb). Essentially, the temperatures that are not enough to break dormancy and start the spring regrowth but still higher than Tb contribute to the cumulative GDD. Therefore, requirement of more AGDD and the delayed flowering in BVL plants are the results of its extreme winter temperatures.

**Table 5:** Alfalfa AGDD requirement to reach the flowering in the spring. This was estimated in Julian days, from the first day of the calendar year, based on two locations JPC and BVL. This shows that alfalfa flowers earlier in 2017 with mild winter temperature than in 2015 with more severe winter

Year	Environment	Flowering Date	TOF	T-max	T-min	AGDD
2015	JPC	13th April	112	12.5	−7	641.1
2017	JPC	27th March	85	18.9	−2.5	587.6
2015	BVL	27th May	146	11.4	−12.6	876.1
2017	BVL	29th April	118	15.5	−8.8	729.6

JPC and BVL refer to the J. Phil Campbell Sr. Research and Education Center (JPC) in Watkinsville and the Georgia Mountain Research and Education Center (BVL) in Blairsville

*T-max* Maximum average temperature (°C) in two winter months (Jan. and Feb.), *T-min* Minimum average temperature (°C) in two winter months (Jan. and Feb.), *AGDD* Accumulated growing degree days from 1st January to the day of early flowering record, the AGDD was obtained by adding growing degree days (GDD) of the effective days

### QTL mapping

#### QTL of timing of flowering (TOF)

In this study, 32 alfalfa chromosomes identified using the GBS SDA SNPs for respective parents (3010 and CW 1010) were used for mapping the phenotypic traits. The genetic maps were relatively dense (~ 1.5 cM/marker) with the female and male linkage maps consisting of 1837 SNPs and 1377 SNPs, respectively. Within 32 homologs of the maternal parent 3010, 13 significant QTL (LOD ≥ 3.0) for flowering time were identified. The QTL were coded as Tof-d1, Tof-d2, …, Tof-d13 to denote the QTL of TOF detected in the ‘dormant’ parent. Because there was nearly one-month difference in TOF between plants in JPC and BVL, we analyzed each environment and year data separately. The most important flowering QTL for 3010 parent, Tof-d7 (*R*^*2*^ = 0.15), was for early flowering (− allele direction) and was detected on homolog 3B with a LOD value of 7.7 (Table 6). Out of the 13 flowering time QTL detected in the 3010 parent, eight QTL showed positive effects on the phenotypic value (+ allele direction) and the remaining five QTL had negative effects on the phenotype, or they are associated with earlier flowering (Table 6). In both environments, for 3010 parents, we detected QTL for both early (−) and delayed (+) TOF. Most of the flowering QTL were identified on homologs of chromosome seven, three and one in the 3010 parent (Table 6). A QTL was also identified on homolog 6D. There were two putative QTL detected on homolog 7B with +ve allele direction (late flowering) in the 3010 parent; however, they were not reported here for being detected at a LOD value of 2.8 which was below the threshold (LOD ≥ 3.0).

**Table 6:** QTL for TOF in alfalfa identified in a pseudo-testcross F1 (3010 x CW 1010) population. The phenotypic data was assessed for two years for TOF in two locations (JPC and BVL)

Parent	QTL code	Chr.	Location/Year	Peak Marker	Peak LOD	R^2^	Allele Dir.	LSI (cM)	Flanking Markers
3010	Tof-d1	1D	JPC/ Π	MRG_14469273	4.4	0.08	–	34.9–36.9	TP56677 - TP66486
3010	Tof-d2	1D	JPC/Π	TP60376	3.3	0.06	–	30.6–31.1	TP60376 - TP41436
3010	Tof-d3	3C	JPC/Π	TP52465	3.5	0.06	+	38.1–44.0	TP37583 - TP72054
3010	Tof-d4	7A	JPC/Π	TP58371	3.3	0.06	+	27.3–30.4	TP58371 - TP2134
3010	Tof-d5	7A	JPC/Π	TP24733	3.8	0.07	+	37.5–38.7	TP55743 - TP34483
3010	Tof-d6	1A	JPC/β	TP85729	3.3	0.06	+	95.1–96.6	TP5699 - TP36877
3010	Tof-d7	3B	JPC/β	TP68861	7.7	0.15	–	42.5–45.9	TP60221 - TP68861
3010	Tof-d8	7A	JPC/β	TP28256	5.1	0.09	+	2.4–6.1	TP28256 - TP80202
3010	Tof-d9	7B	JPC/β	TP3421	4.8	0.09	+	23–27.2	TP9376 - TP3421
3010	Tof-d10	1A	BVL/Π	TP35274	3.3	0.07	+	88.9–91.3	TP52576 - TP995
3010	Tof-d11	1B	BVL/Π	TP23433	3.8	0.09	–	45–49.7	TP23433 - TP66714
3010	Tof-d12	3D	BVL/Π	TP18933	3.4	0.07	–	19.3–24.4	MRG_22559848 - TP66479
3010	Tof-d13	6D	BVL/β	MRG_2402742	3.7	0.08	+	16.2–23.0	TP16313 - TP18699
CW 1010	Tof-n1	5B	JPC/Π	TP11856	4.1	0.07	–	75.3–79.4	TP80448 - TP80460
CW 1010	Tof-n2	6B	JPC/Π	TP3310	7.1	0.14	–	17.2–21.2	TP3310 - TP71145
CW 1010	Tof-n3	6D	JPC/Π	TP48161	4.8	0.09	+	54.8–56.4	MRG_5981048 - TP6188
CW 1010	Tof-n4	6D	JPC/Π	TP49028	5.5	0.10	+	60.1–60.5	TP70280 - TP66860
CW 1010	Tof-n5	6D	JPC/Π, BVL/ β	TP24444	3.8	0.08	+	65.9–66.8	TP64001- TP32647
CW 1010	Tof-n6	7C	JPC/Π, JPC/β	TP44666	8.0	0.16	–	42.9–44.7	TP45002 - TP4972
CW 1010	Tof-n7	7C	JPC/Π, JPC/β	TP54614	4.1	0.07	–	48.4–54.7	TP38417 - TP54614
CW 1010	Tof-n8	7B	JPC/β	TP9019	4.0	0.07	–	23.7–26.1	TP14107 - MRG_9345022
CW 1010	Tof-n9	7B	JPC/β	TP36500	4.7	0.09	–	30.8–31.6	TP36500 - MRG_25777286
CW 1010	Tof-n10	8B	BVL/Π	TP76596	3.6	0.08	–	19.3–31.5	TP76596 - TP75547
CW 1010	Tof-n11	8B	BVL/Π	TP75547	3.1	0.07	–	31.5–38.9	TP75547 - TP25170
CW 1010	Tof-n12	4B	BVL/ β	TP66329	4.6	0.09	–	5.5–12.8	TP88701 - TP57672

JPC and BVL refer to the two locations - the J. Phil Campbell Sr. Research and Education Center (JPC) in Watkinsville and the Georgia Mountain Research and Education Center (BVL) in Blairsville, GA, respectively

The negative allele direction (−) refers to the shorter days to flowering or the QTL responsible for early flowering, and the positive allele direction (+) refers to the QTL responsible for late flowering. The peak and flanking markers given are based on 1- LOD support interval (LSI)

*Π* Year 2015, *β* Year 2017, *Chr.* Chromosome, *Dir.* Direction, *LSI* 1-LOD support interval in cM unit

**Fig. 2:**
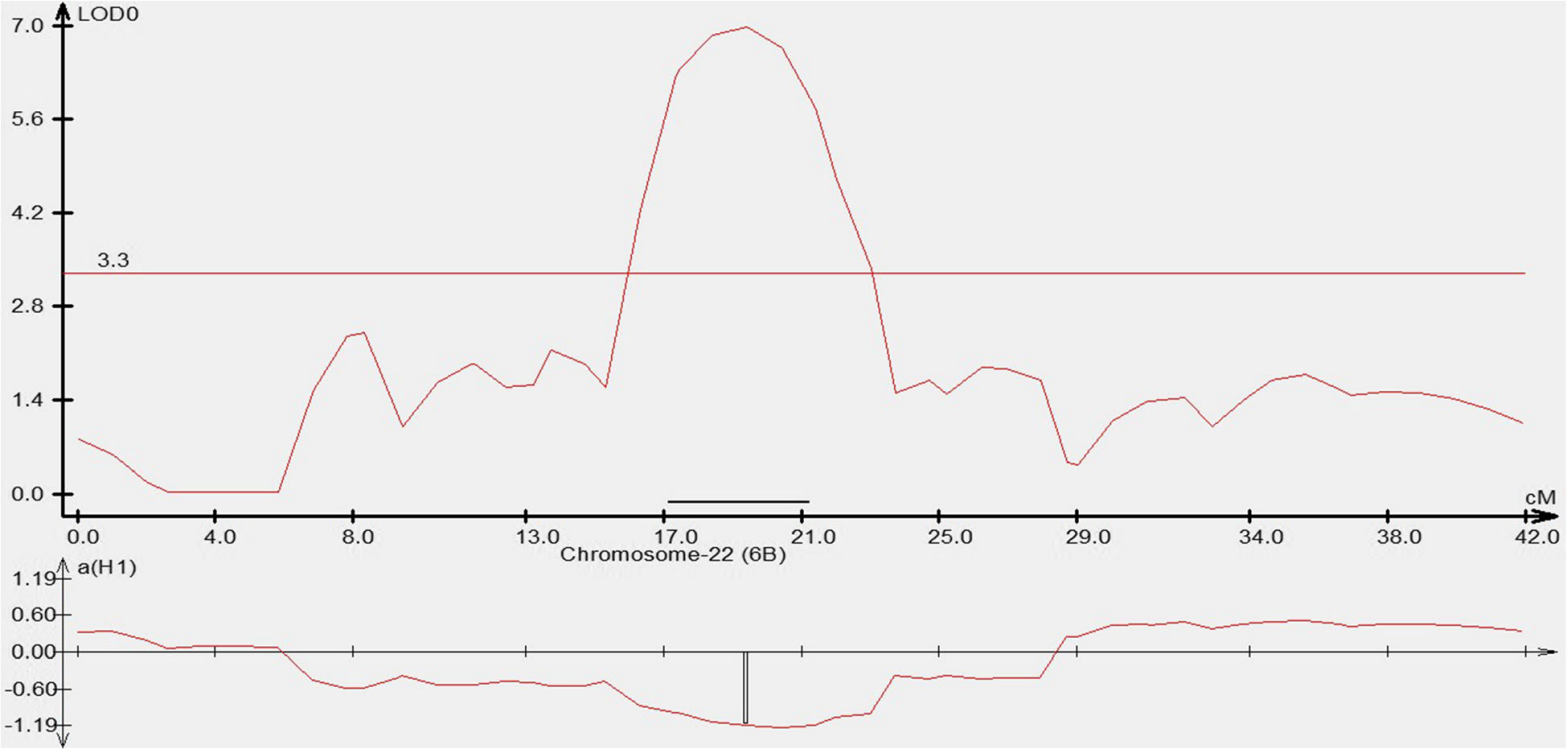
QTL peak for the flowering QTL ‘Tof-n2’ detected on chromosome 6B at LOD = 7.1 for the parent CW 1010. Since the Tof-n2 has an effect in negative direction, the QTL induces early flowering (shorter days to flowering)

For the CW 1010 parent, 12 QTL associated with TOF were identified on different homologs of chromosome four, five, six, seven, and eight (Table 6). The QTL were coded as Tof-n1, Tof-n2, …, Tof-n12 to denote the QTL of TOF for the ‘non-dormant’ parent. Out of 12 QTL, the homologs of chromosome six (Table 6 and Fig. 2) and seven each harbored four QTL. Two other QTL were located on homologs 8B and the remaining two QTL were identified on homologs 4B and 5B (Table 6). The QTL Tof-n6 detected on homolog 7C of CW 1010 explained the highest phenotypic variance (*R*^*2*^ = 0.16). Three flowering QTL Tof-n5, Tof-n6 and Tof-n7 were identified as stable QTL as were detected in more than one environment and/or season. As in QTL of 3010, the flowering QTL of CW 1010 carried both types of alleles (+ and -). Nine QTL were identified for early flowering (− allele direction) and other three QTL were identified for delayed flowering (+ allele direction) (Table 6). The QTL for delayed flowering were detected only on homolog 6D for this parent. The peak and flanking markers of relevant QTL are summarized in Table 6.

#### QTL of SY and CSB

We identified 10 QTL of SY within the homologs of the 3010 parent, and named as SY-d1, SY-d2, …, SY-d10 (Table 7). All QTL detected in the 3010 parent had negative allele effects on the phenotype, suggesting that fall dormant progenies would have reduced biomass yield in the first spring harvest. The QTL SY-d1 detected with LOD = 5.8 explained the highest phenotypic variance (*R*^*2*^ = 0.11) for SY in 3010 parent, however it had a negative effect on SY. The other SY QTL detected in 3010 parent also had negative effects (Table 7). On the genetic linkage maps of CW 1010, we mapped seven different QTL (SY-n1, SY-n2, …, SY-n7) associated with SY (Table 7). The QTL for CW 1010 spring yield were located on homologs 8A, 8B, 8C, 7A and 3D. All QTL detected within the CW 1010 homologs possessed positive effects on SY, indicating that non fall-dormant parent also carries QTL for higher spring yield. The QTL SY-n5 explained the highest phenotypic variance (*R*^*2*^ = 0.13) for SY in CW 1010 parent.

**Table 7:** QTL for spring yield (SY) and cumulative summer biomass (CSB) identified in a pseudo-testcross F1 (3010 x CW 1010) population. The SY phenotypic data was collected for two years and CSB phenotypic data was collected for one year

Parent	QTL Code	Chr.	Location/Year	Peak Marker	Peak LOD	R^2^	Allele Dir.	LSI (cM)	Flanking Markers
3010	SY-d1	3A	JPC/β	TP2592	5.8	0.11	–	56.5–57.1	TP2592 - TP37840
3010	SY-d2	3A	JPC/β	TP83334	4.1	0.08	–	63.5–64.9	TP59541 - TP83334
3010	SY-d3	4D	JPC/β	MRG_4464574	5.1	0.10	–	67.1–70.5	MRG_4464482 - TP43038
3010	SY-d4	7A	JPC/β	TP51377	4.9	0.10	–	76.2–83.2	TP51377 - TP47813
3010	SY-d5	7A	JPC/ф	MRG_10667023	4.1	0.08	–	40.9–43.2	TP30610 - MRG_10666968
3010	SY-d6	7C	JPC/ф	TP66942	3.1	0.06	–	88.8–91.4	TP87634 - TP66942
3010	SY-d7	7D	JPC/ф	TP34947	4.5	0.09	–	33.7–35.8	TP14368 - TP40888
3010	SY-d8	1A	BVL/β	TP46942	3.7	0.07	–	72–72.9	TP72089 - TP46942
3010	SY-d9	1B	BVL/β	MRG_25771949	3.2	0.06	–	76.7–79.7	TP6511 - TP34670
3010	SY-d10	1D	BVL/β	TP1567	3.7	0.07	–	26.4–26.9	TP89308 - TP1567
3010	CSB-d1	3A	JPC/β	TP16385	4.2	0.10	–	0–5.5	TP16385 - TP32175
3010	CSB-d2	4D	JPC/β	TP32956	3.1	0.06	–	103.5–106.8	TP83938 - TP55849
3010	CSB-d3	8C	JPC/β	TP27142	3.5	0.07	–	43.7–46.3	TP66239 - TP27142
CW 1010	SY-n1	3D	JPC/β	TP85451	3.4	0.10	+	5.3–7.6	TP11255 - TP85451
CW 1010	SY-n2	8A	JPC/β	TP45400	3.0	0.06	+	24.7–25.8	TP9008 - TP41903
CW 1010	SY-n3	8B	JPC/β	TP25170	3.0	0.06	+	34.5–44.8	TP25170 - TP86491
CW 1010	SY-n4	8C	JPC/ф	TP27703	4.9	0.12	+	33.3–35.9	TP31047 - TP27703
CW 1010	SY-n5	8C	JPC/ф	TP77807	4.0	0.13	+	51.4–52.5	TP40142 - TP77807
CW 1010	SY-n6	7A	BVL/β	MRG_28464923	4.0	0.07	+	32.4–34	TP57427 - MRG_12020287
CW 1010	SY-n7	7A	BVL/β	TP50516	4.7	0.08	+	40.1–44.2	TP13897 - MRG_4633212
CW 1010	CSB-n1	1C	JPC/β	TP10914	3.5	0.07	+	13.4–17.4	TP11572 - TP42278
CW 1010	CSB-n2	4D	JPC/β	TP83595	3.3	0.06	+	23.2–26.1	TP83595 - TP70955
CW 1010	CSB-n3	5B	JPC/β	TP26255	3.2	0.06	–	34.9–37.5	TP26255 - TP18857

JPC and BVL refer to the two locations - the J. Phil Campbell Sr. Research and Education Center (JPC) in Watkinsville and the Georgia Mountain Research and Education Center (BVL) in Blairsville, respectively

The negative allele direction (−) refers to the low biomass or the QTL responsible for reduced biomass yield, and the positive allele direction (+) refers to the QTL responsible for the higher biomass. The peak and flanking markers given are based on the 1- LOD support interval (LSI)

*β* Year 2017, *ф* Year 2018, *Chr.* Chromosome, *Dir.* Direction, *LSI* 1-LOD support interval in cM unit

Three QTL (CSB-d1, CSB-d2, CSB-d3) associated with CSB were mapped on the maternal (3010) linkage map. The QTL were detected on homologs 3A, 4D and 8C. All these QTL detected for 3010 parent had a negative effect on phenotype. The QTL CSB-d1 explained the highest phenotypic variance (*R*^*2*^ = 0.10) (Table 7). Similarly, three QTL associated with CSB were also detected on homologs 1C, 4D and 5B for CW 1010 parent. The QTL CSB-n1 and CSB-n2 displayed positive effects, whereas a QTL CSB-n3 showed a negative effect on the CSB.

### Identification of potential candidate genes

Several potential candidate genes and corresponding proteins were identified for SNP sequences of the QTL associated with TOF and SY using BLAST_n_ search against Mt4.0, A17 reference genotype pseudomolecules database (Table 8) [18]. Potential candidate genes were declared if sequences were aligned with ≥95% identity as described previously [30]. The sequences of the SNPs in the QTL regions detected in this study, which were obtained from UNEAK pipeline, are provided in the Additional files 1 and 2. The Additional file 1 includes tag sequences of SNPs associated with QTL detected for the maternal parent 3010 and the Additional file 2 includes the SNPs relevant to CW 1010 QTL. However, in this study we included only BLAST result of SNPs in the peak and flanking regions of detected QTL (Table 8). We identified 13 potential candidate genes  associated with 10 different flowering QTL of 3010. Also, nine potential candidate genes associated with six different flowering QTL for CW 1010 were identified (Table 8). Similarly, 10 potential candidate genes were identified for SNPs of eight different SY QTL for 3010, and eight potential candidate genes were identified for six SY QTL for CW 1010. For CSB, we found three potential candidate genes associated with two QTL for 3010 and six potential candidate genes of SNPs of three CSB QTL for CW 1010. The potential candidate genes could be a target for crop improvement of biomass yield through MAS.

**Table 8:** Potential candidate genes identified through BLASTn searchin *M. truncatula* pseudomolecule using tag sequences of SNPs in the regions spanned by QTL for TOF, SY, and CSB mapped on linkage maps of two alfalfa parents 3010 and CW1010

Parent	QTL Code	Markers	Potential Candidate Genes Related Proteins	% Identity	E-value	*M. truncatula* Gene
3010	Tof-d1	TP66486	Myosin motor domain protein and Dil domain protein	98.36	4e-23	Medtr1g070400
3010	Tof-d2	TP60376	P-loop nucleoside triphosphate hydrolase superfamily protein	98.44	9e-25	Medtr1g075200
3010	Tof-d5	TP34483	Pre-mRNA splicing factor-like protein	100.00	2e-26	Medtr7g068630
3010	Tof-d6	TP36877	Chitinase	100.00	2e-26	Medtr1g099320
3010	Tof-d7	TP60221	Hypothetical protein	96.88	4e-23	Medtr8g064300
3010	Tof-d8	TP28256	U6 snRNA-associated-like-Smprotein	100.00	2e-26	Medtr8g058537
3010	Tof-d8	TP80202	Translational activator GCN1-like protein	100.00	2e-26	Medtr7g116425
3010	Tof-d9	TP3421	bZIP transcription factor	100.00	2e-26	Medtr7g088090
3010	Tof-d9	TP9376	Pre-mRNA-splicing factor SLU7-like protein	100.00	2e-26	Medtr7g096940
3010	Tof-d10	TP35274	Carbohydrate-binding X8 domain protein	96.88	4e-23	Medtr1g084820
3010	Tof-d10	TP995	Importin-like protein	96.43	1e-18	Medtr7g021500
3010	Tof-d11	TP23433	Exocyst complex component sec15B	98.44	9e-25	Medtr1g050505
3010	Tof-d12	TP53864	RNA-binding (RRM/RBD/RNP motif) family protein	95.3	2e-21	Medtr3g027140
3010	SY-d1	TP2592	LOB domain protein	96.88	4e-23	Medtr3g452660
3010	SY-d2	TP83334	Nudix hydrolase-like protein	98.44	9e-25	Medtr3g437740
3010	SY-d4	TP51377	RS2-interacting KH protein, putative	100.00	2e-26	Medtr7g013700
3010	SY-d4	TP47813	Peptide/nitrate transporter	98.44	9e-25	Medtr7g010820
3010	SY-d6	TP66942	Transcription factor	95.31	7e-21	Medtr7g092510
3010	SY-d7	TP14368	RING/U-box protein	96.88	4e-23	Medtr7g056183
3010	SY-d8	TP46942	Succinyl-CoA ligase [ADP-forming] subunit beta	100.00	2e-26	Medtr1g069645
3010	SY-d9	TP6511	Plastid transcriptionally active protein	100.00	2e-26	Medtr1g079525
3010	SY-d9	TP34670	Alpha/beta hydrolase family protein	96.72	2e-21	Medtr1g088470
3010	SY-d10	TP89308	Lon protease S16 carboxy-terminal proteolytic domain protein	95.24	7e-21	Medtr1g083990
3010	CSB-d2	TP32956	1-aminocyclopropane-1-carboxylate oxidase-like protein	100.00	2e-26	Medtr4g099390
3010	CSB-d2	TP83938	Phosphatase 2C family protein	98.39	1e-23	Medtr4g118340
3010	CSB-d3	TP27142	C2H2-type zinc finger protein, putative	96.88	4e-23	Medtr4g057230
CW 1010	Tof-n1	TP628	Aluminum activated malate transporter family protein	100	2e-26	Medtr5g014310
CW 1010	Tof-n1	TP47971	F-box/RNI/FBD-like domain protein	100	2e-26	Medtr5g012840
CW 1010	Tof-n5	TP24444	Cytochrome P450 family protein	96.88	4e-23	Medtr1g116890
CW 1010	Tof-n5	TP64001	Granule bound starch synthase	98.39	1e-23	Medtr6g012380
CW 1010	Tof-n5	TP32647	Pentatricopeptide (PPR) repeat protein	95.31	2e-21	Medtr6g022140
CW 1010	Tof-n6	TP45002	Group 1 family glycosyltransferase	100.00	2e-26	Medtr7g067340
CW 1010	Tof-n7	TP54614	Pre-mRNA splicing factor-like protein	98.44	9e-25	Medtr7g068630
CW 1010	Tof-n8	TP14107	Det1 complexing ubiquitin ligase	95.31	2e-21	Medtr7g091260
CW 1010	Tof-n11	TP25170	Polyol/monosaccharide transporter 1	100.00	2e-26	Medtr4g090600^a^
CW 1010	SY-n1	TP11255	CCCH-type zinc finger protein, putative	98.44	9e-25	Medtr3g464260
CW 1010	SY-n2	TP41903	Armadillo repeat only protein	100.00	2e-26	Medtr4g073830
CW 1010	SY-n3	TP25170	Polyol/monosaccharide transporter 1	100.00	2e-26	Medtr4g090600^a^
CW 1010	SY-n3	TP86491	Pentatricopeptide (PPR) repeat protein	100.00	2e-26	Medtr8g106950
CW 1010	SY-n4	TP27703	ATP-dependent helicase BRM	100.00	2e-26	Medtr8g030550
CW 1010	SY-n5	TP77807	Trafficking protein particle complex subunit-like protein	100.00	2e-26	Medtr8g027700
CW 1010	SY-n7	TP50516	Pyruvate decarboxylase	95.31	7e-21	Medtr7g069500
CW 1010	SY-n7	TP13897	ARM repeat protein	96.88	4e-23	Medtr7g075940
CW 1010	CSB-n1	TP10914	XS domain protein	98.44	9e-25	Medtr1g492940
CW 1010	CSB-n1	TP11572	Octicosapeptide/phox/Bem1p family protein	98.44	9e-25	Medtr1g109470
CW 1010	CSB-n1	TP42278	Hypothetical protein	96.88	4e-23	Medtr1g079830
CW 1010	CSB-n2	TP70955	60 kDa inner membrane protein	98.44	9e-25	Medtr4g107330
CW 1010	CSB-n3	TP26255	Endoribonuclease E-like protein	96.88	4e-23	Medtr5g030900
CW 1010	CSB-n3	TP18857	Homeobox domain protein	96.88	4e-23	Medtr2g014490

The Tof-n11 is a flowering QTL and SY-n3 is a QTL for summer yield identified for the same CW 1010 parent

TOF, SY, and CSB refer to the timing of flowering, spring yield, and cumulative summer biomass, respectively

^a^ potential candidate genes identified for corresponding SNP sequences of both QTL (Tof-n11 and SY-n3)

## Discussion

### Segregation of F1 for TOF

This study explored the analysis of QTL for flowering time and biomass yield using a pseudo-testcross mapping population derived from two alfalfa cultivars, CW 1010 (♂) and 3010 (♀) with contrasting FD and WH. It is worth noting that mapping in an F1 population reflects the variation within each of the parents rather than between them. Whether the two parents exhibit obvious variation in the trait may not therefore be critical to mapping the trait as the random segregation of chromatids may generate new combinations of loci that will translate in variation in the progeny. Different allele combinations at the loci underlying the trait may result in similar flowering time in the parents, but their segregation in the progeny will result in much more variation in flowering time. The mapping population used in this study displayed a near normal distribution of flowering time phenotype even though the difference between the parents was not large (Fig. 1). The genotypes in northern GA location (BVL) flowered about a month later than in JPC and subsequently the genotypes in BVL were harvested nearly a month later. Therefore, the influence of the environment on phenotype or G x E was obvious (Table 1). The TOF and SY exhibited a range of heritability per dataset indicating the impact of the environment. However, flowering time had mostly a higher H^2^ than the spring biomass yield, except in BVL017 dataset, which indicated the impact of various environmental factors on yield and flowering time of alfalfa in various degrees (Table 1).

Identification of the multiple QTL controlling flowering time suggests that TOF is a polygenic trait in alfalfa. Moreover, QTL for early and delayed flowering were detected in both parents, suggesting that the flowering time is defined by various combinations of alleles at the loci controlling the trait. The maternal parent 3010 flowered slightly earlier than the CW 1010 parent, despite that it carried a higher number of QTL (8 out of total13) for delayed flowering than early flowering (5 out of total 13), suggesting that some of these QTL may have a bigger effect than others. Some of the important flowering QTL detected on 3010 genome were for early flowering, such as Tof-d7 (*R*^*2*^ = 0.15) and Tof-d11 (*R*^*2*^ = 0.09) (Table 6). Similarly, in CW 1010 genome, we found the early flowering QTL such as Tof-n6 (*R*^*2*^ = 0.16) and Tof-n2 (*R*^*2*^ = 0.14) which have larger effects than the delayed flowering QTL such as Tof-n3 (*R*^*2*^ = 0.09) and Tof-n4 (*R*^*2*^ = 0.10). The non-dormant parent CW 1010 carried a higher number of early flowering QTL compared to the dormant parent 3010. Therefore, both parents could pass any combination of alleles in the QTL for TOF to their progeny. However, since CW 1010 carried a higher number of early flowering QTL, the chance of early flowering alleles being present in the progenies from the non-dormant parent is higher. Predicting FD based on TOF on alfalfa seems unrealistic, at least in this population, because of the lack of strong phenotypic correlation between the traits, and the presence of both early and delayed flowering loci in both parents. Also, the results indicate that flowering time manipulation in both dormant and non-dormant alfalfa is possible without affecting their dormancy levels, which is considered important for alfalfa adaptation to specific latitudes.

The presence of transgressive segregants on either side of flowering time might be the result of different allele combinations during meiotic random segregation and recombination in the parents carrying QTL alleles for both early and late TOF. Assuming each heterozygous parent carries a single QTL for TOF that delays flowering and the QTL have similar effects, progenies that carry both QTL could be flowered later than each of the parent if the effect of the two alleles is additive, while the parents would have similar flowering times. Likewise, if a progeny carries an early flowering QTL allele from a parent and a delayed flowering QTL allele with similar effect from another parent, then that progeny would have intermediate TOF.

### Correlation among traits

Phenotypic correlations among agronomic and adaptation traits in alfalfa, such as SY, TOF, seasonal dormancy and WH reported in the study can be valuable for production management and trait manipulation to improve yield. For instance, developing a winter-hardy non-dormant alfalfa cultivar with early spring regrowth and flowering would be ideal to extend the growing period in early winter, and to harvest fresh forage earlier in the spring in regions with mild winters. Previously, we described the possibility of simultaneous improvement of FD and WH [31]. This study revealed that the manipulation of flowering time, used frequently as a management indicator of harvest time, is possible in both dormant and non-dormant alfalfa. However, the correlations presented here were based on phenotypic data and hence may not necessarily represent the exact magnitude and sign of genetic correlations among these traits. Falconer and Mackay (1996) suggested that the genetic correlations among traits may not be fully estimated using only the phenotypic correlations [32]. Therefore, the correlations presented in this study need to be validated by determining the genetic correlations using available markers for this population for effective MAS for multiple traits. Further, the AGDD requirement for flower initiation appears to be environment specific at least in this population which is not exceptional.

### Evolutionarily conserved TOF QTL

We detected 25 flowering QTL including 13 for 3010 parent and 12 for CW 1010 parent. Some of these QTL were consistently expressed in multiple locations and/or years (Tof-n5, Tof-n6, and Tof-n7). Some flowering QTL detected on both parental linkage maps were consistent with previously reported flowering QTL in *M. truncatula* [1] indicating that these QTL are possibly evolutionarily conserved and supporting the validity of QTL detection. The flowering QTL Tof-n10 (19.3–31.5 cM) detected on 8B homolog corresponded to the genomic location of *M. truncatula* QTL on chromosome 8 (12–23 cM) [1], and both induced early flowering. The flowering QTL Tof-d5 on sub-genome 7A for 3010 parent, which spanned 37.5–38.7 cM at 1-LOD support interval (LSI) and 36.8–39.0 cM at 2-LSI, corresponded to the genomic region on chromosome 7 with a flowering QTL of *M. truncatula* reported previously [1]. These two flowering QTL also exhibited similar function of inducing delayed flowering. Similarly, The stable alfalfa flowering QTL Tof-n7 (48.4–54.7 cM) on 7C for the CW 1010 parent was identified in a corresponding genomic position of an *M. truncatula* flowering QTL (47–65 cM) on corresponding chromosome 7 [1], and both conferred early flowering. The QTL Tof-n6 corresponded to the genomic region on chromosome 7 of *M. truncatula* flowering QTL, but with opposite phenotypic effect. Furthermore, we identified other novel QTL on different homologs of chromosome 7 for 3010 and CW 1010, such as Tof-d4, Tof-d8, Tof-d9, Tof-n8 and Tof-n9 (Table 6). Therefore, chromosome 7 and its homologs in alfalfa are very important genomic sites for flowering QTL as in *M. truncatula* [3]. Chromosome 7 of *M. truncatula is* also recognized for the presence of copies of flowering locus T (*FT*) [1, 3]. Several other novel QTL of TOF detected in this study on various alfalfa chromosomes add valuable genomic resources for the molecular manipulation of TOF in this complex polyploid species. Since we observed QTL for both early flowering and delayed flowering, the trait can be improved in either direction. Early flowering may be desirable for early spring cutting in environments with mild winter and delayed flowering may be a choice in the regions where early spring frost compromises the plant regrowth.

### Novel QTL for SY and CSB

A total of 17 QTL associated with SY in alfalfa (ten for 3010 and seven for CW 1010) were detected in this study. Robins et al. [20] mapped alfalfa biomass production on genetic linkage maps constructed using restriction fragment length polymorphism (RFLP) and simple sequence repeat (SSR) markers using single marker analysis. They further mapped forage yield, plant height, and regrowth on the same genetic maps [33]. However, both mapping studies were carried on only eight alfalfa linkage groups, unlike the sub-genome level mapping in this study. They detected markers associated with yield mainly on linkage groups 3, 4, 7 and 8. We also detected QTL of SY in some homologs of these chromosomes for either parent (Table 7). The markers with positive effects on the spring biomass, especially detected in the non-dormant parent, would be an essential target for enhancing alfalfa biomass. Additionally, the potential QTL obtained for CSB such as CSB-n1 and CSB-n2, which have positive effects on biomass would be useful for enhancing yield of the cool season alfalfa in the summer months. Nevertheless, exploring the low effect magnitude QTL may not be ideal and the situation can be better handled by deploying genomic selection (GS) with adequate size training population and markers to improve alfalfa quantitative traits. The stable, conserved and high effect QTL and their markers reported in this study can be directly used for MAS for alfalfa improvement.

###  Potential candidate genes

Among the potential candidate genes (22 for TOF, 18 for SY and 9 for CSB) identified using *M. truncatula* genome, several showed near perfect to perfect homology (~ 100% identity) (Table 8). A *M. truncatula* gene ‘Medtr4g090600’ was detected as potential candidate for SNPs of both the QTL of flowering (Tof-n11) as well as SY (SY-n3), indicating possible overlapping of the pathways for TOF and SY in alfalfa. The number of candidate genes could be increased if we had scanned an entire sequence of the QTL regions. This is because the genetic maps used in this study are dense and mostly consist of several SNPs under QTL regions. Relaxing stringency of LOD support interval (LSI), such as using 2-LSI instead of 1-LSI, could also enhance the numbers of SNPs associated with the traits. Functions of the potential candidate genes identified in this study using *M. truncatula* reference genome can also be searched in other model plant species. For instance, a potential gene identified for TOF QTL (Tof-d9) in this study, known as a bZIP transcription factor (Table 8), was previously reported as a candidate gene for flowering time in *M. truncatula* [1] and Arabidopsis [34]. The sequences given in Additional files 1 and 2 would be useful for comparative genomics analysis of the relevant genomic regions of the timing of flowering and yield traits. Therefore, detailed study of potential candidate genes and their roles in biological pathways relevant to phenotypic variations would be valuable to identify the candidate genes for the corresponding traits.

## Conclusions

In this study, we mapped stable and novel QTL associated with important agronomic and adaptation traits on relatively saturated genetic maps, adding valuable genomic resources for alfalfa improvement. This study showed that alfalfa fall dormancy and spring flowering time do not correlate to an extent that one can predict using the other in F1 plants of heterozygous parents with contrasting dormancy. However, the phenotypic correlations observed for alfalfa traits under different environments would be valuable for both crop management and genetic improvement. As the QTL identified in this study are linked to a set of GBS SNPs in their sub-genomes, homologous gene search can be expanded to other databases of *M. truncatula* and alfalfa genomes available at the diploid level to identify candidate genes. The trait associated QTL and SNPs that were stably present and have higher effect size can be used to accelerate alfalfa breeding and achieve higher genetic gain. However, validation of the reported QTL in diverse genetic backgrounds and multiple environments is recommended. Also, genomic selection (GS) could be an effective approach for enhancing these quantitative traits in alfalfa because most of the QTL identified had relatively low effect size.

## Methods

### Mapping population development and phenotyping

Plant material (F1 progenies, parents, and standard checks), experimental design, and testing sites were also described previously [31, 35]. The seeds of alfalfa cultivars 3010 (BrettYoung, Winnipeg, Manitoba, Canada) and CW 1010 (Alforex Seeds, Woodland, CA, USA) with fall dormancy values 2 and 10, respectively, were germinated in the greenhouse at the University of Georgia (UGA), Athens. The greenhouse was maintained at 18 h. light and 6 h. dark. A population of 184 F1 progenies was obtained by hand crossing (3010 ♀ x CW 1010 ♂) and their parentage was confirmed using five SSR markers. The final mapping population included 181 F1 plants with good quality and informative markers out of total 184 plants. The plants were established at two environments in Georgia, JPC and BVL, using a RCBD design with three replications in August 2014. Four clones of each F1 genotype and two parents prepared through stem cuttings were transplanted in a single row. The check cultivars for FD and WH were directly seeded in row plots. Spring TOF was recorded every 3 days from the beginning of March until the appearance of at least one flower in all four clones of each genotype. Flowering data were taken during the spring seasons of the years 2015 and 2017. The TOF was recorded in Julian calendar days (i.e. January 1st = 1).

The first fresh biomass harvest of the spring (SY) was collected in each of the years 2017 and 2018 using a swift forage harvester (Swift Machine and Welding Ltd., Saskatchewan, Canada). Three subsequent summer cuts after the spring harvest were recorded in 2017 in both environments. The cumulative summer biomass (CSB) data consisted of the sum of the dry weights of the three summer cuts. Because of the frequent rainfall in the BVL area in 2018, biomass harvests were more delayed and spread compared to the Watkinsville location. The dry matter percent was estimated from bulked samples selected randomly from all three replications. The samples were dried in a convection oven at 60 °C for 3 days. The phenotypic data for both TOF and yield was fitted to the generalized linear model to obtain least square (LS) means of each trait [31]. Each dataset for each year and environment was analyzed separately because of genotype by environment and year (G x E x Y) interactions. Correlations between various phenotypic traits were estimated using the Proc Corr procedure in SAS 9.4 [36]. Timing of flowering recorded for 2 years (TOF015 and TOF017), SY recorded for two subsequent years (SY017 and SY018), FD recorded for 2 years (FD015 and FD017), seasonal dormancy assessed in the winter of 2017 (WD017), and WH data for 2 years (WH016 and WH017) were analyzed and Pearson correlation coefficients (r) were determined (Tables 3 and 4). The correlation between CSB and other traits was also evaluated for 2017. To validate the correlations obtained in the F1 population, the check cultivars were also compared to the same variables.

### G x E and heritability

For each of the trait, we initially fitted a generalized linear model as described in our previous work [31];

$$ \mathrm{Trait}\ \mathrm{value}=\mathrm{genotype}\ \left(\mathrm{g}\right)+\mathrm{environment}\ \left(\mathrm{e}\right)+\mathrm{block}\ \left(\mathrm{r}\right)+\mathrm{genotype}\ast \mathrm{environment}\ \left(\mathrm{g}\mathrm{e}\right)+\mathrm{Error}\ \left(\upvarepsilon \right) $$

where the phenotypic variations were taken as the cumulative effects of genotype, environment, their interactions and residual errors. However, because of significant (*P* < 0.05) G x E and the differences of data collection periods in two locations, we estimated the trait values (least square means) for each individual experiment separately using PROC GLM in SAS 9.4 [34] as;

$$ \mathrm{Trait}\ \mathrm{value}=\mathrm{genotype}\ \left(\mathrm{g}\right)+\mathrm{block}\ \left(\mathrm{r}\right)+\mathrm{Error}\ \left(\upvarepsilon \right) $$

Broad sense heritability (H^2^) of TOF was estimated using variance components σ^2^_g_, σ^2^_r,_ and σ^2^_ε_, with the formula;

$$ {\mathrm{H}}^2=\frac{{\upsigma^2}_{\mathrm{g}}}{\frac{{\upsigma^2}_{\mathrm{g}}+{\upsigma^2}_{\mathrm{r}}+{\upsigma^2}_{\upvarepsilon}}{\mathrm{r}}} $$

Where, σ^2^_g_, σ^2^_r_ and σ^2^_ε_ are the variance components of genotype, block and residual error, respectively. The number of blocks is denoted by r. To estimate the variance components, we fitted the linear mixed model with R (https://www.R-project.org/) with an R package *lme4* and a function *lmer*. Subsequently, the variance components were estimated using the restricted maximum likelihood (REML) approach considering all factors as random.

The effect of accumulated growing degree days (AGDD) on spring flowering time in alfalfa was assessed at the base temperature of five degrees Celsius as described in a previous report [37]. The GDD calculation for two seasons (2015 and 2017) and two environments beginning from January 1 of the year to the spring flowering date was summarized (Table 5). The AGDD was estimated using the formula;

$$ \mathrm{AGDD}={\sum}_{i=0}^n\left(\frac{\mathrm{Tmax}\hbox{-} \mathrm{Tmin}}{2}\right)-\mathrm{Tb} $$

Where, Tmax, Tmin and Tb refer to the maximum temperature (°C), the minimum temperature (°C) and the base temperature (5 °C), respectively [38]. The base temperature (Tb) is a minimum threshold temperature below which no growth occurs. Temperature data were obtained from the UGA weather station at Watkinsville-UGA, Oconee County, Georgia (http://weather.uga.edu/index.php?content=calculator&variable=CC&site=WATUGA.).

### QTL mapping and potential candidate gene identification

The methods used for linkage map construction and QTL mapping were described previously [31]. DNA extraction for the progeny and the parents was carried out using the CTAB method with some modifications [39]. The single dose allele (SDA) SNP markers that segregated in 1:1 (1/2 Aaaa:1/2 aaaa) and polymorphic to either one of the parents were retained from the set of raw SNP markers discovered by the GBS method. Of 184 F1 plants, quality markers were identified for 181 genotypes and hence the traits were mapped on 181 genotypes. The parental linkage maps contained the SNPs polymorphic in CW 1010 parent and the maternal linkage maps were constructed with SDA SNPs polymorphic to 3010 parent. The 32 linkage groups for each of the two parents were aligned with the *M. truncatula* reference genome (Mt4.0v2) using BLAST search. Four alfalfa homologs were grouped and randomly assigned for each *M. truncatula* chromosome. Then, the QTL were mapped using LS means of TOF and SY as phenotypic values. QTL mapping was conducted using the composite interval mapping (CIM) method in Windows QTL Cartographer version 2.5 (http://statgen.ncsu.edu/qtlcart/WQTLCart.htm.). Comparative analysis of genomic regions was performed using tag sequences of the SNPs in the QTL peak and flanking regions. The sequences were subjected to BLAST search against *M. truncatula* genome (Mt4.0) (http://www.medicagohapmap.org/tools/blastform) and the best BLAST hit outputs were viewed on GBrowse (http://www.medicagohapmap.org/fgb2/gbrowse/mt40/?). The potential candidate genes for the QTL regions were identified and their relevant functions were searched in the literatures (Table 8).

## Additional files

Additional file 1: Sequences of SNPs obtained from UNEAK. The sequences given below were of the SNPs associated with the alfalfa flowering time and yield QTL detected for maternal parent 3010. Two variant alleles for each SNP were denoted as ‘query’ and ‘hit’. (DOCX 24 kb)

Additional file 2: Sequences of SNPs obtained from UNEAK pipeline. The sequences given below were of the SNPs associated with alfalfa flowering time and yield QTL detected for paternal parent CW 1010. Two variant alleles for each SNP were denoted as ‘query’ and ‘hit’. (DOCX 19 kb)

## Data Availability

The raw reads from GBS work can be found in the NCBI SRA, which can be accessed at https://www.ncbi.nlm.nih.gov/sra/SRP150116

## Abbreviations

AGDD: Accumulated growing degree days
CSB: Cumulative summer biomass
FD: Fall dormancy
G x E: Genotype by environment
GBS: Genotyping-by-sequencing
GS: Genomic selection
MAS: Marker-assisted selection
NGS: Next generation sequencing
SDA: Single dose allele
SY: Spring yield
TOF: Timing of flowering
WH: Winter hardiness
WS: Winter survival
