# Correction to: QTL mapping of flowering time and biomass yield in tetraploid alfalfa (*Medicago sativa* L.)

**DOI:** 10.1186/s12870-019-2020-7

**Published:** 2019-10-28

**Authors:** Laxman Adhikari, Shiva Om Makaju, Ali M. Missaoui

**Affiliations:** Institute of Plant Breeding, Genetics and Genomics and Department of Crop and Soil Sciences, The University of Georgia, Athens, GA USA

**Correction: BMC Plant Biology (2019) 19:359**

**https://doi.org/10.1186/s12870-019-1946-0**

In the article [1], in ‘Methods’ section and ‘G x E and heritability’ subsection, there is an error in the formula of heritability (H^2^).

The correct formula should be

$$ {\mathrm{H}}^2=\frac{\upsigma^2\mathrm{g}}{\sigma^2\mathrm{g}+\frac{\upsigma^2\mathrm{r}+{\upsigma}^2\upvarepsilon}{\mathrm{r}}} $$

The original publication has been corrected.
