# Supplementary material for: QTL mapping of flowering time and biomass yield in tetraploid alfalfa (Medicago sativa L.)
Source: BMC Plant Biol. 2019 Aug 16;19:359. doi: 10.1186/s12870-019-1946-0 (PMC6697951; doi:10.1186/s12870-019-1946-0)
Supplement: Supplementary file 1 — Sequences of SNPs obtained from UNEAK. The sequences given below were of the SNPs associated with the alfalfa flowering time and yield QTL detected for maternal parent 3010. Two variant alleles for each SNP were denoted as ‘query’ and ‘hit’. (DOCX 24 kb) [file 12870_2019_1946_MOESM1_ESM.docx]

Sequences of SNPs obtained from UNEAK. The sequences given below were of the SNPs associated with the alfalfa flowering time and yield QTLs detected for maternal parent 3010. Two variant alleles for each SNP were denoted as ‘query’ and ‘hit’.

**Chromosome: 1A**

**QTL: Tof-d6**

>TP5699_query

CAGCAAGTGCACCAAGATGGCAAGTACTGTCACACCTGTGCTTACTCGAAAGGTGATATTTCAT

>TP5699_hit

CAGCAAGTGCACCAGGATGGCAAGTACTGTCACACCTGTGCTTACTCGAAAGGTGATATTTCAT

>TP85729_query

CTGCTTACTCATACAACTAAAATATTCAATTTACTAACGATCTTGTGTCGTGCTAAATGATTGA

>TP85729_hit

CTGCTTACTCATACAACTAAAATATTCAATTTACTAACGATCTTGTGTCGTGCTAAATGATTGC

>TP36877_query

CAGCTCCTCAATGTCCAATTCCAGATAAATTTTTGGGCACTGCACTTGAAACAGGTCTTTTTGA

>TP36877_hit

CTGCTCCTCAATGTCCAATTCCAGATAAATTTTTGGGCACTGCACTTGAAACAGGTCTTTTTGA

**QTL: Tof-d10**

>TP52576_query

CTGCACAAAACTGAATCAAACAACAACGAATCTACTATGGCCGTGAAGCTCCTGAATCCGCACC

>TP52576_hit

CTGCACGAAACTGAATCAAACAACAACGAATCTACTATGGCCGTGAAGCTCCTGAATCCGCACC

>TP35274_query

CAGCTATTCTTCTTGCTTCTAAATATTTGGATACTCTTTTACTAAATCGAGTTACTTCTATTGA

>TP35274_hit

CAGCTATTCTTTTTGCTTCTAAATATTTGGATACTCTTTTACTAAATCGAGTTACTTCTATTGA

>TP995_query

CAGCAAACAACCTTGCTATTAAAACACAGATCTAACGAAAAATTTGAATAGTTTTCTTCTTCTT

>TP995_hit

CAGCAAACAACCTTGCTATTAAAACACAGATCTAACGAAAAATTTGAATAGTTTTCTTCTTGTT

**QTL: SY-d8**

>TP72089_query

CTGCCTTTTCAAAATCAGTGTTCTCTAATTGCATATAGCTGTTACTAAGTTGTCTCATAGTTAA

>TP72089_hit

CTGCCTTTTCAAAATCATTGTTCTCTAATTGCATATAGCTGTTACTAAGTTGTCTCATAGTTAA

>TP46942_query

CTGCAAAGGTCGTTGATGGTTTGGCTCCTAAAGTGGCTGATAGAAATCAATCAATTGAACAAGT

>TP46942_hit

CTGCAAAGGTTGTTGATGGTTTGGCTCCTAAAGTGGCTGATAGAAATCAATCAATTGAACAAGT

**Chromosome: 1B**

**QTL: SY-d9**

>TP6511_query

CAGCAATCATATCATACATAACTTGAGAAACCCCGGTTGAGTCACGGTTCCTAGCCCGGTTCAT

>TP6511_hit

CAGCAATCATATCATACATAACTTGAGAAACTCCGGTTGAGTCACGGTTCCTAGCCCGGTTCAT

>TP67204_query

CTGCCATTGATGCAAATGTTCATGCGCCAAATGAAACGATATTATAACACTTGTATCTTTCTCC

>TP67204_hit

CTGCCATTGATGCAAATGTTCATGCGCCAAATGAAACGATATTATAGCACTTGTATCTTTCTCC

>TP34670_query

CAGCTATATAAAGACAGTAAAATATATAGGTCAATTTATGTATGTTGTATAAGAGCGCATCCAA

>TP34670_hit

CAGCTATATAAAGACAGTAAAATATATAGGTCAATTTATGTATGTTGTATAAGAGCGCATCTAA

**QTL: Tof-d11**

>TP23433_query

CAGCCCAACTATGCGGCATCCCTTGCCGATCAATTGAAAGGCCTCAGCCAGATTTAAGTGCAAA

>TP23433_hit

CAGCCCAACTATGTGGCATCCCTTGCCGATCAATTGAAAGGCCTCAGCCAGATTTAAGTGCAAA

>TP66714_query

CTGCCATCTTTTTTACGTAAGGTGATGTTCTTGGGTCAGTGGCTAATTCTTCATCCACTTTGAG

>TP66714_hit

CTGCCATCTTTTTTACGTAAGGTGATGTTCTTGGGTCGGTGGCTAATTCTTCATCCACTTTGAG

**Chromosome: 1D**

**QTL: Tof-d1**

>TP56677_query

CTGCAGATACACTTGATAAGTTGATATTGGCTTCGAGTTTATGAAAATGATTTCCGCCGAACCT

>TP56677_hit

CTGCAGATACACTTGATAAGTTGATATTGGCTTGGAGTTTATGAAAATGATTTCCGCCGAACCT

>TP66486_query

CTGCCATCAAAATTCAAGCCTATACACGTGGAAGATTACCCAGAAAATGCTACTCTGAAATAAA

>TP66486_hit

CTGCCATCAAAATTCAAGCCTATACACGTGGAAGATTATCCAGAAAATGCTACTCTGAAATAAA

**QTL: Tof-d2**

>TP60376_query

CTGCATCATCTCTACTAGTGGCGGCTCTAAACAAAGGGCGAGATGTGATCATGGACGGTACCTT

>TP60376_hit

CTGCATCATCTCTACTAGTGGCGGCTCTAAACAAAGGGCGGGATGTGATCATGGACGGTACCTT

>TP41436_query

CAGCTTCAAAGGCTGTGATTGTTGAGGCAGATGCTTTTAAGGAGAGTGATGTTATATATAAAGC

>TP41436_hit

CAGCTTCAAAGGCTGTGGTTGTTGAGGCAGATGCTTTTAAGGAGAGTGATGTTATATATAAAGC

**QTL: SY-d10**

>TP89308_query

CTGCTTTTATATCCACTCACTCACCAATAAATACCATTTGTTTATTAGAAATTTTAGTCATGTA

>TP89308_hit

CTGCTTTTATATCTACTCACTCACCAATAAATACCATTTGTTTATTAGAAATTTTAGTCATGTA

>TP1567_query

CAGCAAAGCTGTAGAGAAACTTCTCAAAGACAAATGGGCTCAGACTGTCTCATCACAGCCATCA

>TP1567_hit

CAGCAAAGCTGTAGAGAAATTTCTCAAAGACAAATGGGCTCAGACTGTCTCATCACAGCCATCA

**Chromosome: 3A**

**QTL: SY-d1**

>TP2592_query

CAGCAACAAGAACATGAGCTTCAGATGGGTGGTGGATATCAACTTACTCCACAAGAGTATTTGC

>TP2592_hit

CAGCAACAAGAACATGAGCTTCAGATGGGTGGTGGATATCAACTTACTCCACAGGAGTATTTGC

>TP37840_query

CAGCTCTTAGTACTCATTATAGCATCAAAGATTCCAGTTTTCTTCCACAGTGATTCATATTTGG

>TP37840_hit

CAGCTCTTAGTACTCATTATAGCATCAAAGATTCCAGTTTTTTTCCACAGTGATTCATATTTGG

**QTL: SY-d2**

>TP59541_query

CTGCATATAGAAAAATTACCATCAGCCTCTACCAAAACAACCCCGTGGAACAAAAACTGCTCAA

>TP59541_hit

CTGCATATAGAAAAATTACCATCAGCTTCTACCAAAACAACCCCGTGGAACAAAAACTGCTCAA

>TP83334_query

CTGCTGATTTATTGCATAAAGCTGGGGACATTTCTGACTCCACAAGGTATATTGCTCTTTCATC

>TP83334_hit

CTGCTGATTTATTGCATAAAGCTGGGGACATTTCTGACTCCACAAGGTATATTGTTCTTTCATC

**QTL: CSB-d1**

>TP16385_query

CAGCATCCTTCAAAATGGAAATGAAGGCTCCTTAGCAAATGCTTGTGACACGAACAACTACCAA

>TP16385_hit

CAGCATCCTTCAAAATGGAAATGAAGGCTCCTTAGCAGATGCTTGTGACACGAACAACTACCAA

>TP32175_query

CAGCGTTTTCAAAAGAAGAAGAAAGTGCATTAGTTCCTTATAAATAGATTTGCCAAGTGGGGGA

>TP32175_hit

CAGCGTTTTCAAAAGAAGAAGAAAGTGCATTAGTTCCTTATAAATAGATTTGCCAAGTGGGTGA

**Chromosome: 3B**

**QTL: Tof-d7**

>TP60221_query

CTGCATCAGAGAGTTTCTTCTACAACTTTGGAACGACTTCATCAATTGGTCTCCTTAAATTCCC

>TP60221_hit

CTGCATCAGAGAGTTTCTTCTACAACTTTGGAACGACTTCATCAATTGGTCTCCTTTAATTCCC

>TP68861_query

CTGCCCTTTTGTGGAGCATGTGGAAACATAGAAACTTATAGCTCTGGAAAGGCGCGTGTCGGAA

>TP68861_hit

CTGCCCTTTTGTGGAGCATGTGGAAACATAGAAACTTATAGCTCTGGAAAGGCGCGTTTCGGA

**Chromosome: 3C**

**QTL: Tof-d3**

>TP37583_query

CAGCTCTCCCTTCTCTGGATTCTTCTATCTCTCCCACTGAATATTCTTCACAGCACCTTCACTT

>TP37583_hit

CAGCTCTCCCTTCTCTGGATTCTTCTATCTCTCCCTCTGAATATTCTTCACAGCACCTTCACTT

>TP52465_query

CTGCAATTTTATGACAGGAGAATGCAAGTGCAGAATCATTTGCTAGCCTTTTGTGACAAATTAG

>TP52465_hit

CTGCAATTTTATGACAGGAGAATGCAAGTGCAGAATCATTTGCTGGCCTTTTGTGACAAATTAG

>TP72054_query

CTGCCTTTGTTAAAATAATCTACAACTAGTTTCACACCCATCTCAAAATCTAAATTTGTAAGTT

>TP72054_hit

CTGCCTTTGTTAAAATAATCTACAACTAGTTTCGCACCCATCTCAAAATCTAAATTTGTAAGTT

**Chromosome: 3D**

**QTL: Tof-d12**

>TP30040_query

CAGCGCATGGTAAGAACATGAGATTTTACTTTATCCTTCCAGCATTTATTTCTTAAACACTACA

>TP30040_hit

CAGCGCCTGGTAAGAACATGAGATTTTACTTTATCCTTCCAGCATTTATTTCTTAAACACTACA

>TP53864_query

CTGCACCAGCACCATTAATGTGCAATGCAAATGGCCTCAACTGACGACCAGCTACTGTTTCACT

>TP53864_hit

CTGCACCAGCACCATTAATGTGCGATGCAAATGGCCTCAACTGACGACCAGCTACTGTTTCACT

>TP18933_query

CAGCATTTACATCCTCATGGTTAAATGAAACAAAATAGGAACAATAAAACACAGGAAAATAAAT

>TP18933_hit

CAGCATTTACATCCTCATGGTTAAATGAAACAAAATAGGAACAATCAAACACAGGAAAATAAAT

>TP66479_query

CTGCCATATTTATGCACAGTTTGGTAGAGGTTTGGTCCTGCTATTAATCGCTTCACAACTTATT

>TP66479_hit

CTGCCGTATTTATGCACAGTTTGGTAGAGGTTTGGTCCTGCTATTAATCGCTTCACAACTTATT

**Chromosome: 4D**

**QTL: SY-d3**

>TP44049_query

CAGCTTTACTTCATTGAGTTTGTTTGTACTTATGTCAAGGAGAATCTCTTATTCTCTTATCCAT

>TP44049_hit

CAGCTTTACTTCATTGAGTTTGTTTGTACTTATGTCAAGGAGTATCTCTTATTCTCTTATCCAT

>TP43038_query

CAGCTTGAGATATATGACTGTTAACATCTGTTTGAAAAAATTAAAAAGCAATATAAAATATAAA

>TP43038_hit

CAGCTTGAGATATATGACTGTTAACATTTGTTTGAAAAAATTAAAAAGCAATATAAAATATAAA

**QTL: CSB-d2**

>TP83938_query

CTGCTGGTCATCCCTAACCCGTTGCAATACTGTAGACACTGATTTCGATGTTTACTTCCCTGCT

>TP83938_hit

CTGCTGGTCATCCCTAACCCGTTGCAATACTGTTGACACTGATTTCGATGTTTACTTCCCTGCT

>TP32956_query

CAGCTAATGACGAATGACACGTTCATCAGTGTTAATCACCGGGTGTTGTCAAGGAATATAGGTC

>TP32956_hit

CAGCTAATGACGAATGACACGTTCATCAGTGTTGATCACCGGGTGTTGTCAAGGAATATAGGTC

>TP55849_query

CTGCAGAAACAGAAGATTTGTTGCAACTTTTTGTCTAAACGTTCCTAACTTACTCAAACAGCAT

>TP55849_hit

CTGCAGAAACAGAAGATTTGTTGCAACTTTTTGTCTAAACGTTCCTAACTTACTCAATCAGCAT

**Chromosome: 6D**

**QTL: Tof-d13**

>TP16313_query

CAGCATCCGTACCCAAATTCACATTAGAATCCATACCTTTGTATTTTCAAAGTATAAACCCTAA

>TP16313_hit

CAGCATCCGTACCCAAATTCACATTAGAATCCATACCTTTGTGTTTTCAAAGTATAAACCCTAA

>TP18699_query

CAGCATTGCTATGAGCGTGTTTGTTTACGGTTACTCTTGGCATTCCAGAATGCATTTTTGCGGC

>TP18699_hit

CAGCATTGCTATGAGCGTGTTTGTTTACGGTTACTCTTGGCATTCTAGAATGCATTTTTGCGGC

**Chromosome: 7A**

**QTL: Tof-d4**

>TP58371_query

CTGCAGTTACACTATGATCCTTGATATTATATTGCAGATAATTGCAGATATATGTTGTTGAGGT

>TP58371_hit

CTGCAGTTACACTATGATCCTTGATATTATATTGCAGATAATTGCAGATTTATGTTGTTGAGGT

>TP34795_query

CAGCTATCAGCAAATACCAGGCCTTTATAAAACACAATATTCGTGAAAGTAAATTCATTTGTGT

>TP34795_hit

CAGCTATCAGCAAATACCATGCCTTTATAAAACACAATATTCGTGAAAGTAAATTCATTTGTGT

>TP2134_query

CAGCAAATTACTAGCTAAAGAGTTTAGCTTGAGAGTTACCACGGAATAACTACTGAATTAGCCG

>TP2134_hit

CAGCAAATTACTAGCTAAAGAGTTTAGCTTGAGAGTTACCGCGGAATAACTACTGAATTAGCCG

**QTL: Tof-d5**

>TP55743_query

CTGCACTTTTAACCATTATCATTATGCATTCATTTTTTTTCTTGTTATATTTTGTCACTTCAAA

>TP55743_hit

CTGCACTTTTAATCATTATCATTATGCATTCATTTTTTTTCTTGTTATATTTTGTCACTTCAAA

>TP24733_query

CAGCCCTGTATAGAGAATGTGCTTGAGTCGTGGTTCCAGAACTATAATTCAAAAAATAGCCATG

>TP24733_hit

CAGCCCTGTATAGAGAATGTGCTTGAGTCGTGGTTCCAGAACTATAATTCAAAAAATATCCATG

>TP34483_query

CAGCTATAACATGGGGATCATGCTCACATTTCTTTAGGGCATCCATACTCTTGCTTTTACGTTG

>TP34483_hit

CAGCTATAACATGGGGATCATGCTCGCATTTCTTTAGGGCATCCATACTCTTGCTTTTACGTTG

**QTL: Tof-d8**

>TP28256_query

CAGCCTTCCAGTTATATAAAAAATAAAGTTATCTAATCAATGTCACCCTTTTCAGATCGCAAGC

>TP28256_hit

CAGCCTTCCAGTTATATAAAAAATAAAGTTATCTAATCAATGTTACCCTTTTCAGATCGCAAGC

>TP80202_query

CTGCTCCAGAGCGTTCAACATTACTATTATCAGACTTAAGAGTTTCAAACAACCATGGGACAAG

>TP80202_hit

CTGCTCCAGAGCGTTCCACATTACTATTATCAGACTTAAGAGTTTCAAACAACCATGGGACAAG

**QTL: SY-d4**

>TP51377_query

CTGCAATGAAGGCAACCCAAATGTTGCAATTAGGCGGCTAGGGGTGGACCGCAATGAAAGCGGA

>TP51377_hit

CTGCAATGAAGGCAACCCAAATGTTGCAATTAGGCGGCTAGGGGTGGACCGCAATGAAGGCGGA

>TP47813_query

CTGCAACAAAAACTTGACAAAACCTAGGAAGAGGGTTTCCAGCAGGTTTAAAGTATCTATATTT

>TP47813_hit

CTGCAACAAAAACTTGGCAAAACCTAGGAAGAGGGTTTCCAGCAGGTTTAAAGTATCTATATTT

**QTL: SY-d5**

>TP30610_query

CAGCGGAGCAAGAAAGTAGGGTTAAAGAAAAAGACAAGTTGCAAAGAGGATTATGAACAAATGA

>TP30610_hit

CAGCGGAGCAAGAAATTAGGGTTAAAGAAAAAGACAAGTTGCAAAGAGGATTATGAACAAATGA

**Chromosome: 7B**

**QTL: Tof-d9**

>TP9376_query

CAGCACCCTCATCATTTTGGTCGCCCTTCTCCAATCTCTTCAGCTGTTGTTCCTTCAAGAACTT

>TP9376_hit

CAGCACCCTCATCATTTTGGTCGCCCTTCTCCAGTCTCTTCAGCTGTTGTTCCTTCAAGAACTT

>TP3421_query

CAGCAACCACAACAACAACAACAGCATCAAAATCATCAGAATAATATGATGACTGGTTTTGCAG

>TP3421_hit

CAGCAACCACAACAACAACATCAGCATCAAAATCATCAGAATAATATGATGACTGGTTTTGCAG

**Chromosome: 7C**

**QTL: SY-d6**

>TP87634_query

CTGCTTGATCAAAATTTTAACATATAAACAACCAAGTAATTTCCCAAAACTTAGTGCAGGCAAG

>TP87634_hit

CTGCTTGATCAAAATTTTAACATATAAACAACCAAGTAATTTCCCAAAACTTAGTGCAGGCATG

>TP66942_query

CTGCCATGGTCCCATAATTTTGGATTATTTGACTTAGAATCTATAAAAAAATCCTTTTCTCAAT

>TP66942_hit

CTGCCATGGTCCCATAATTTTGGATTGTTTGACTTAGAATCTATAAAAAAATCCTTTTCTCAAT

**Chromosome: 7D**

**QTL: SY-d7**

>TP14368_query

CAGCATAAAATGGTTGAGCTCGGTCTTGCGGGAAAGGGGGATAACCAAATGGCACAGGTGGGTT

>TP14368_hit

CAGCATGAAATGGTTGAGCTCGGTCTTGCGGGAAAGGGGGATAACCAAATGGCACAGGTGGGTT

>TP34947_query

CAGCTATGACACAAGGAATTTGTGAGCTATTGTGCGTAAAAGAAGTGTAAGAAGATTTGAAGAT

>TP34947_hit

CAGCTATGACACAAGGAATTTGTGAGCTATTGTGCGTAAAGGAAGTGTAAGAAGATTTGAAGAT

>TP40888_query

CAGCTTACACTCTTCCTTGAAAGAGTCGAGGTTGTTGCTGAAAACAAAGATAATTAAGTGGGAT

>TP40888_hit

CAGCTTACACTCTTCCTTGAAAGAGTTGAGGTTGTTGCTGAAAACAAAGATAATTAAGTGGGAT

**Chromosome: 8C**

**QTL: CSB-d3**

>TP66239_query

CTGCCATAAATTCATACTCTCATATTGATAAATATAAATTGTTCTAATGATCGATATAAAGAAG

>TP66239_hit

CTGCCATAAATTTATACTCTCATATTGATAAATATAAATTGTTCTAATGATCGATATAAAGAAG

>TP27142_query

CAGCCTCGGCCACAGTCTTCATCACATGATTCGCAATCAGTTAACCTGGTTGCGAATAGTGATG

>TP27142_hit

CAGCCTCGGCCACAGTCTTCATCACATGATTCGCAATCAGTTAATCTGGTTGCGAATAGTGATG
