# Supplementary material for: QTL mapping of flowering time and biomass yield in tetraploid alfalfa (Medicago sativa L.)
Source: BMC Plant Biol. 2019 Aug 16;19:359. doi: 10.1186/s12870-019-1946-0 (PMC6697951; doi:10.1186/s12870-019-1946-0)
Supplement: Supplementary file 2 — Sequences of SNPs obtained from UNEAK pipeline. The sequences given below were of the SNPs associated with alfalfa flowering time and yield QTL detected for paternal parent CW 1010. Two variant alleles for each SNP were denoted as ‘query’ and ‘hit’. (DOCX 19 kb) [file 12870_2019_1946_MOESM2_ESM.docx]

Sequences of SNPs obtained from UNEAK pipeline. The sequences given below were of the SNPs associated with alfalfa flowering time and yield QTLs detected for paternal parent CW 1010. Two variant alleles for each SNP were denoted as ‘query’ and ‘hit’.

**Chromosome: 1C**

**QTL: CSB-n1**

>TP11572_query

CAGCAGAATTAGGAGCCATGGACTGAGACGGATGGTGGATCTGGGAGTAAGTGACATACTGCTG

>TP11572_hit

CAGCAGGATTAGGAGCCATGGACTGAGACGGATGGTGGATCTGGGAGTAAGTGACATACTGCTG

>TP10914_query

CAGCACTTGCCACATCTTCATATGATTCGTCATTAATCGTATGCTTGTTCACATTATATCCATG

>TP10914_hit

CAGCACTTGCCACATCTTCATATGATTCGTCATTAATCGTATGCTTGTTCTCATTATATCCATG

>TP42278_query

CAGCTTCTAATATTGCGAAAAAATAATCACTAACGCCAGACTTAACACTTCCTGCCTCAATCAA

>TP42278_hit

CAGCTTCTAATATTGCGAAAAAATAATCGCTAACGCCAGACTTAACACTTCCTGCCTCAATCAA

**Chromosome: 3D**

**QTL: SY-n1**

>TP11255_query

CAGCAGAACATCCAGAAACAGCACAACGAAGAGCGGTAGCCCTATCTGAGCCGCACTCTCGGTT

>TP11255_hit

CAGCAGAACATCCAGAAACAGCACAATGAAGAGCGGTAGCCCTATCTGAGCCGCACTCTCGGTT

>TP85451_query

CTGCTGTTTTTCGGATAGGCTGGGACTATGTGTGCTGGATTTTCCTGTCAGTTTTGGGGAGCCC

>TP85451_hit

CTGCTGTTTTTCGGATAGGCTGGGACTATGTGTGCTGGTTTTTCCTGTCAGTTTTGGGGAGCCC

**Chromosome: 4B**

**QTL: Tof-n12**

>TP88701_query

CTGCTTTCATCAGTTCATCAAATGTATCAAAGTTTAGAATTTTGAAATGTTCTTATGTGGCTAG

>TP88701_hit

CTGCTTTCATCAGTTCATCAAATGTATCGAAGTTTAGAATTTTGAAATGTTCTTATGTGGCTAG

>TP66329_query

CTGCCATACATATAAGCTTGAGCCCAGTTTTAGAATTTTTCTTTGATAAAGGGTTTTAAAATTG

>TP66329_hit

CTGCCATATATATAAGCTTGAGCCCAGTTTTAGAATTTTTCTTTGATAAAGGGTTTTAAAATTG

>TP57672_query

CTGCAGGTTTACCAATTTATTTTAAAAGTTGGCCGAGTCATCTGGTTCACACCGGTTTTCCATA

>TP57672_hit

CTGCAGGTTTACCAATTTATTTTAAAAGTTGGCCGAGTCATCTGGTTCACGCCGGTTTTCCATA

**Chromosome: 4D**

**QTL: CSB-n2**

>TP83595_query

CTGCTGGATATAATCATTTTTGTAGAAATATGTCCACGACACCGGATCAGGGTTTCGATAAGAT

>TP83595_hit

CTGCTGGATATAATCATTTTTGTAGAAATATGTCCATGACACCGGATCAGGGTTTCGATAAGAT

>TP70955_query

CTGCCTCTTACAAAGAGGAAAAAACCTCATAGATCGAAGACGCAACCCTTCTTTCACCTATATT

>TP70955_hit

CTGCCTCTTACAAAGAGGAAAAAACCTCATAGATCGAAGATGCAACCCTTCTTTCACCTATATT

**Chromosome: 5B**

**QTL: Tof-n1**

>TP80448_query

CTGCTCCGACGAAAGGTAGTAGGTCCGGTGGAGGTAGTGGAAGCGGGTCACAGATGGTGGATTC

>TP80448_hit

CTGCTCCGACGAAAGGTGGTAGGTCCGGTGGAGGTAGTGGAAGCGGGTCACAGATGGTGGATTC

>TP11856_query

CAGCAGAGCCACCATCCACGTGTAAACAATAATACATGAAAGAATCAGGGACTCACATGCATCA

>TP11856_hit

CAGCAGAGCCACCATCCACGTGTAAACAATAATACATGAAAGAATCAGGGACTCACATGCGTCA

>TP628_query

CAGCAAAAGGAAGTGCCTCAGAGAATTCAAGACTAGTGATAGCAATTTTACTCAACTGAGGTCT

>TP628_hit

CAGCAAAAGGAAGTGCCTCAGAGAATTCAAGACTAGTGATAGCAATTTTACTCAACTGGGGTCT

>TP47971_query

CTGCAACAAGCATCCTCTCCAAGAAATGGAACCAACTATGGCTCTCAGTTCTCACTCTTGATTT

>TP47971_hit

CTGCAACAAGCATCCTCTCCAAGAAATGGAACCAACTATGGCTCTCCGTTCTCACTCTTGATTT

>TP80460_query

CTGCTCCGATTGTACGATTTTGACCCAGATAGTGAAGGGAAATCTCTCGTATTTTAAATACGAC

>TP80460_hit

CTGCTCCGATTGTACGATTTTGACCCAGATAGTGAAGGGAAATCTCTCGTATTTTAACTACGAC

**QTL: CSB-n3**

>TP26255_query

CAGCCTAAGGACTATTGCCCCAAAAGAAATAAAGCTACTGAAGCATTTGCTAAACATCATAGAC

>TP26255_hit

CAGCCTAAGGACTATTGCCCCAAAAGAAATAAAGCTACTGAAGCATTTGCTAAACATCATAGAT

>TP18857_query

CAGCATTGTCTCTCAGGCTTTTTGAGTTCGATTCATCCATCAACTACGTAAAGCTTGAGAGACT

>TP18857_hit

CAGCATTGTCTCTCAGGTTTTTTGAGTTCGATTCATCCATCAACTACGTAAAGCTTGAGAGACT

**Chromosome: 6B**

**QTL: Tof-n2**

>TP3310_query

CAGCAACATTATTGTTTAAGGCATCACAACTCTGCGAACTAAAAGTATGTGCATTCTGAAATGA

>TP3310_hit

CAGCAACATTATTGTTTAAGGCATCACAACTCTGCGAACTAAAATTATGTGCATTCTGAAATGA

>TP71145_query

CTGCCTGCAATTGGAGTGCAAAAGTAAAAGGTTAAGAACATTTATAAATCTCAAGTTGCTTGTG

>TP71145_hit

CTGCCTGCAATTGGAGTGCAAATGTAAAAGGTTAAGAACATTTATAAATCTCAAGTTGCTTGTG

**Chromosome: 6D**

**QTL: Tof-n3**

>TP48161_query

CTGCAACACTCTCAACATCGACATCATCAACAGCGTGATCATTATTGGATGACCTTTCCGAACC

>TP48161_hit

CTGCAACACTCTCAACATCGACATCATCAACAACGTGATCATTATTGGATGACCTTTCCGAACC

>TP6188_query

CAGCAATAGGGAGTGTACTTGTACTTGGAGAATCCAAACCAAATGCTCCTTCAACACCGTGGAC

>TP6188_hit

CAGCAATAGGGAGTGTGCTTGTACTTGGAGAATCCAAACCAAATGCTCCTTCAACACCGTGGAC

**QTL: Tof-n4**

>TP70280_query

CTGCCTATCCACCCGAACTCAATGTTTTTGTGGACAAAAGGAAGGTGTTTAAAGTTGAGGTCAG

>TP70280_hit

CTGCCTATCCAGCCGAACTCAATGTTTTTGTGGACAAAAGGAAGGTGTTTAAAGTTGAGGTCAG

>TP49028_query

CTGCAACTCCATCAATGATTTGTCGATATCACCACTGATATAAAATTTATTGTCATCATCGCTT

>TP49028_hit

CTGCAACTCCATCAATGATTTGTCGATATCACCACTGATATAAAATTTATTGTTATCATCGCTT

>TP66860_query

CTGCCATGCGACCTCCTTTTCCTCACCATCGCTTCCATGGCTTCACCTTTCTATCTCCCATCAA

>TP66860_hit

CTGCCATGCGACCTCCTTTTCCTCACCATCGCTTCCATGGCTTCACCTTTCTCTCTCCCATCAA

**QTL: Tof-n5**

>TP64001_query

CTGCCAAATACCAATGGTGAAATTCGTAGCATGTACTCCACCTTTGTAGTTTGATCAACATGAA

>TP64001_hit

CTGCCAAATACCAATGGTGAAATTCTTAGCATGTACTCCACCTTTGTAGTTTGATCAACATGAA

>TP24444_query

CAGCCCGTGACATGTTCAAAAACAACGATTCAGCTCTTGCAGGAAGGAAGGTTTATCAGGCCAT

>TP24444_hit

CAGCCCGTGACATGTTCAAAAACAACGATTCAGCTCTTGCAGGAAGGAAGGTTTATGAGGCCAT

>TP41673_query

CAGCTTCACTTCACAATTCTCAAATCGATTCTTCAACAATCCATTCACTCCAAAATTCCCCCAA

>TP41673_hit

CAGCTTCACTTCACAATTCTCAAATCGATTCTTCAACAATCGATTCACTCCAAAATTCCCCCAA

>TP32647_query

CAGCTAACGTAAAGCACAGTGCTTGCATTGTTGATCTTTTAGGACGTGCAGGAAGGCTGGAGGA

>TP32647_hit

CAGCTAACGTAAAGCACAGTGCTTGCATTGTTGATCTTTTAGGACGTTCAGGAAGGCTGGAGGA

**Chromosome: 7A**

**QTL: SY-n6**

>TP57427_query

CTGCAGGGGGCAGAAGACTCTTTGCCAATGGAAGCGCGGCAACAGCACCTTTCTGAGGGTCAGA

>TP57427_hit

CTGCAGGGGGCAGAAGACTCTTTGTCAATGGAAGCGCGGCAACAGCACCTTTCTGAGGGTCAGA

**QTL: SY-n7**

>TP13897_query

CAGCAGTGTGTTACTGATGAAGATAATCCTTACCTGAGAGAATGGGGCTTATGGTGTGTAAGGA

>TP13897_hit

CAGCAGTGTGTTACTGATGAAGATAATCCTTACCTGAGAGAATGGGGCTTATGGTGTGTTAGGA

>TP50516_query

CTGCAAGTTTGGTGGAATCATGGTGGCACTGTGAGATTGTTGAAGCTTCAAAGGCTAGTTTTTG

>TP50516_hit

CTGCAAGTTTGGTGGAATCATGGTGGCACTGTGAGATTGTTGAATCTTCAAAGGCTAGTTTTTG

**Chromosome: 7B**

**QTL: Tof-n8**

>TP14107_query

CAGCAGTTGAGTTAGGCCACTATATGGTGATGGATGAGTATTGCACATTCATGTGCATACCTAC

>TP14107_hit

CAGCAGTTGAGTTAGGCCACTATATGGTGTTGGATGAGTATTGCACATTCATGTGCATACCTAC

>TP9019_query

CAGCACCACCAGCGGCGGACAAAATCAGCAGTTTACGGATGCGATTCTGAGTGGGATTCTCTCT

>TP9019_hit

CAGCACCACCAGCGGCGGACAAAATCAGCAGTTTACGGATGCGATTCTGAGTGGGATTCTGTCT

**QTL: Tof-9**

>TP36500_query

CAGCTCCAGCCATGATCTTCAATCTGGAATCCCAATCTAGTTGAATTCGTCCAGTTTCACTTGT

>TP36500_hit

CAGCTCCAGCCATGATCTTCAATCTGGAATCCCAATCTGGTTGAATTCGTCCAGTTTCACTTGT

**Chromosome: 7C**

**QTL: Tof-n6**

>TP45002_query

CAGCTTTTCCTATGGTTTCTCTCAACTCTTTATCGAACAAAAGGGGTTTCAATTTACTCAAGCA

>TP45002_hit

CAGCTTTTCCTATGGTTTCTCTCAACTCTTTGTCGAACAAAAGGGGTTTCAATTTACTCAAGCA

>TP44666_query

CAGCTTTGCCACACTATGTTGATAAATTCTTTTAATCAAATTTACATTTGCAAACTTGCGAGTA

>TP44666_hit

CAGCTTTGCCACACTATGTTGATAAATTCTTTTAATCAAATTTACATTTGCAAACTTGTGAGTA

>TP4972_query

CAGCAAGCACCCAAGAAAGAAGAGGACGAGTCCCTGATGACAAAACCTATTGAACCACAAGAGG

>TP4972_hit

CAGCAAGCACCCAAGAAAGAAGAGGACGAGTCCCTGATGACAAAGCCTATTGAACCACAAGAGG

**QTL: Tof-n7**

>TP38417_query

CAGCTGAAGGACCTTCCCAAAAAAATGGAGGTAATACATTTTATGAGAACATTCAATAATCAGC

>TP38417_hit

CAGCTGAAGGACCTTCCGAAAAAAATGGAGGTAATACATTTTATGAGAACATTCAATAATCAGC

>TP54614_query

CTGCACGAGCTTTACTCAGCCCACTCATCTCCTCTTCAAGATTAGCAAGAGAAAGCCAAAGGGG

>TP54614_hit

CTGCACGAGCTTTACTCAGCCCACTCATCTCCTCTTCAAGATTAGCAAGAGAGAGCCAAAGGGG

**Chromosome: 8A**

**QTL: SY-n2**

>TP9008_query

CAGCACCACCAATCCTGTTAATGACAATCAGACAATTATTAAGTTCTATAAAGGTGTTGATATA

>TP9008_hit

CAGCACCACCAATCCTGTTAATGACAATCATACAATTATTAAGTTCTATAAAGGTGTTGATATA

>TP45400_query

CTGCAAAAACAAAGATCATCTCAAAGGTCTCATTAGCTGGAAAGTCCAACAAAGCAGTAACTGG

>TP45400_hit

CTGCAAAAACAAAGATCATCTCAAAGGTCTCATTAGCTGGAAAGTCTAACAAAGCAGTAACTGG

>TP41903_query

CAGCTTCATTGGTTTCATTGGCACGTGACAATGATCGTTACGGAAAACTAATTATAGAAGAAGG

>TP41903_hit

CAGCTTCATTGGTTTCATTGGCACGTGACAATGATCGTTACGGAAAACTAATTCTAGAAGAAGG

**Chromosome: 8B**

**QTL: Tof-n10**

>TP76596_query

CTGCTACCAATTTTAAACTATTTAAAACACCGAATTTTTCATCATCATCAATCAGTACCACCAC

>TP76596_hit

CTGCTACCAATTTTAAACTATTTAAATCACCGAATTTTTCATCATCATCAATCAGTACCACCAC

>TP75547_query

CTGCTAAATTTGAGAGTTCGTATGACTAAAATATGTAATTTATACCTTGAGAATAAGAATCAGG

>TP75547_hit

CTGCTAAATTTGAGAGTTTGTATGACTAAAATATGTAATTTATACCTTGAGAATAAGAATCAGG

**QTL: Tof-n11**

>TP25170_query

CAGCCGATCGAATCTCCAATGGAAAGATCTCACTTGTCACCAACCAACCAAGAGGACCCCATGA

>TP25170_hit

CAGCCGATCGAATCTCCAATGGAAAGATCTCACTTGTCACCAACCAACCAAGAGGGCCCCATGA

**QTL: SY-n3**

>TP25170_query

CAGCCGATCGAATCTCCAATGGAAAGATCTCACTTGTCACCAACCAACCAAGAGGACCCCATGA

>TP25170_hit

CAGCCGATCGAATCTCCAATGGAAAGATCTCACTTGTCACCAACCAACCAAGAGGGCCCCATGA

>TP86491_query

CTGCTTCATGGGGAGCTCTACTTGGTGCAAGCCGGATTCATGGCAATACTAAACTGGGCGAGAA

>TP86491_hit

CTGCTTCATGGGGAGCTCTACTTGGTGCAAGCCGGATTCATGGCAATACTGAACTGGGCGAGAA

**Chromosome: 8C**

**QTL: SY-n4**

>TP31047_query

CAGCGGGCGACAGATGTTAAGAAACAGAAGGAATCAAAGCAGAAAGAAAGGAGTAAGTTTGAGG

>TP31047_hit

CAGCGGGTGACAGATGTTAAGAAACAGAAGGAATCAAAGCAGAAAGAAAGGAGTAAGTTTGAGG

>TP27703_query

CAGCCTGCTTTGGGAATTCATTCACAACAACAAGCTAAGATGGGAATGTTAAACCCTGCATCTG

>TP27703_hit

CAGCCTGCTTTGGGAATTCATTCGCAACAACAAGCTAAGATGGGAATGTTAAACCCTGCATCTG

**QTL: SY-n5**

>TP40142_query

CAGCTGTCTTGGGACTCCAATCCGAGCTGGTTATGGAGGGTTAATCCGCAGAAAAAAAAAAAAA

>TP40142_hit

CAGCTGTCTTGGGACTCCGATCCGAGCTGGTTATGGAGGGTTAATCCGCAGAAAAAAAAAAAAA

>TP77807_query

CTGCTAGTTAACTTCATCAACCTCTTCCAGATCTGTAAGCAATTGACGCACAATTGCCCCATAT

>TP77807_hit

CTGCTTGTTAACTTCATCAACCTCTTCCAGATCTGTAAGCAATTGACGCACAATTGCCCCATAT
